# Mitochondrial Ferritin Overexpression Attenuates Ferroptosis and Mitochondrial Dysfunction by Reducing VDAC1 to Relieve MI/RI‐Induced Damage

**DOI:** 10.1111/jcmm.70650

**Published:** 2025-06-23

**Authors:** Yong Yuan, Xiuqi Wang, Huaihuan Xu, Lanxiang Liu, Jichun Liu, Songqing Lai, Huang Huang

**Affiliations:** ^1^ Department of Cardiovascular Surgery, The First Affiliated Hospital, Jiangxi Medical College Nanchang University Nanchang Jiangxi China; ^2^ Department of Cardiovascular Surgery, the Second Affiliated Hospital, Jiangxi Medical College Nanchang University Nanchang Jiangxi China; ^3^ Jiangxi Provincial Key Laboratory of Basic Pharmacology, Jiangxi Medical College Nanchang University Nanchang Jiangxi China

**Keywords:** ferroptosis, ischaemia/reperfusion injury, mitochondrial ferritin, VDAC1

## Abstract

Ischaemic cardiomyopathy is becoming one of the most prevalent cardiovascular diseases among the global elderly population. However, the underlying molecular mechanisms remain incompletely understood. Our previous study demonstrated that VDAC1 plays a significant role in MI/RI. Furthermore, FTMT plays a pivotal role in iron metabolism. However, the precise molecular functions of VDAC1 and FTMT in MI/RI remain to be elucidated. In vitro H9c2 cells A/R and in vivo SD rat MI/RI models were constructed. The present study reports that VDAC1 levels were increased and FTMT levels were decreased in A/R. The overexpression of VDAC1 resulted in an exacerbation of the A/R‐induced injury, characterised by an increase in oxidative stress, a reduction in the GSH/GSSG ratio, the formation of reactive oxygen species, elevated levels of lipid peroxidation, and the deposition of iron. In contrast, FTMT overexpression reversed these alterations and mitigated mitochondrial dysfunction by downregulating VDAC1, PTGS2 levels, upregulating GPX4 levels, inhibiting MPTP over‐opening and stabilising MMP. Additionally, knockdown of VDAC1 alleviated A/R‐induced ferroptosis. In vivo experiments showed that overexpression of FTMT improved cardiac function in rats, as evidenced by the reduction of MI/RI‐induced serum CK‐MB, LDH and Fe^2+^ content and the shrinkage of myocardial infarction area. Moreover, HE, DHE staining and TEM observations showed that the overexpression of FTMT ameliorated MI/RI‐induced myocardial tissue and mitochondrial damage. Furthermore, the overexpression of FTMT was found to inhibit MI/RI‐induced ferroptosis. In general, our study is the first to demonstrate that FTMT overexpression alleviates ferroptosis and mitochondrial dysfunction by regulating VDAC1, thereby reducing MI/RI injury.

## 1 Introduction

Ischaemic cardiomyopathy (ICM) is becoming a prevalent cardiovascular disease among the global elderly population. Acute myocardial infarction, the principal form of ICM, continues to represent the predominant cause of mortality/disability, exerting a considerable burden on society [1, 2]. Although rapid haemodynamic restoration by pharmacological thrombolysis, percutaneous coronary intervention, and surgical intervention remains an effective means of treating AMI, the sudden recovery of haemodynamics may further exacerbate additional myocardial tissue damage or even infarction in patients with AMI. This phenomenon is known as myocardial ischaemia/reperfusion injury (MI/RI) [3, 4]. The physiological mechanisms of MI/RI are complex and varied, involving inflammatory response, oxidative stress, autophagy, apoptosis and necroptosis [5, 6]. However, there is still no effective strategy to prevent and treat MI/RI.

As a novel form of programmed cell death (PCD), ferroptosis has gradually become a research focus in the biomedical field since it was first proposed by Dixon's team in 2012 [7]. Prior research has indicated that reactive oxygen species (ROS) burst, lipid peroxidation and iron deposition are key to promoting ferroptosis [8]. The current literature indicates that ferroptosis plays a role in the pathogenesis of various cardiovascular diseases, particularly MI/RI [9]. Inhibition of ferroptosis has been shown to effectively attenuate MI/RI‐induced injury [10]. Consequently, ferroptosis may be employed as a prospective therapeutic strategy. Nevertheless, the precise molecular mechanism of ferroptosis in MI/RI remains unclear.

Altered mitochondrial morphology is a key feature of ferroptosis, encompassing mitochondrial shrinkage, loss of ridges, and even rupture of the outer mitochondrial membrane (OMM) [11, 12]. The voltage‐dependent anion channel (VDAC), located on the OMM, voltage‐dependent anion channel 1 (VDAC1) isoform is the most abundant of the VDAC clamp and functions as a bidirectional gate, regulating the exchange of metabolites and ions between the mitochondria and the cytoplasm [13, 14, 15]. In addition, mitochondria are a major target for the onset of MI/RI‐induced ferroptosis. Recent studies have demonstrated that VDAC1 activation is associated with MI/RI [16, 17]. Our previous research demonstrated that resveratrol and tanshinone IIA reduced VDAC1 protein levels and inhibited MI/RI (or A/R)‐induced ferroptosis in the myocardium after MI/RI (or A/R) [18, 19]. However, the precise role of VDAC1's contribution to mitochondrial function remains largely unknown.

Mitochondrial ferritin (FTMT) is a ferritin located on mitochondria [20]. Previous studies have reported that FTMT is highly homologous to heavy‐chain ferritin. FTMT, in addition to being a proprietary mitochondrial target for iron storage, has an enzymatic activity to oxidise ferrous ions to ferric ions. Furthermore, the distribution of FTMT is highly tissue‐specific, occurring only in organs with high oxygen metabolism, such as the brain, testis and heart, while it is hardly expressed in the largest iron storage organ (liver) [21, 22, 23, 24]. This indicates that the principal function of FTMT may be to safeguard specific tissues from damage by preventing the accumulation of free iron and oxidative stress. As far as the current study proposes that FTMT may exert a protective effect in stroke by regulating iron homeostasis, ferroptosis and apoptosis [25, 26, 27]. However, the mechanism through which VDAC1‐FTMT acts in mediating ferroptosis in MI/RI remains unclear.

In general, our study is the first to demonstrate that FTMT overexpression alleviates ferroptosis and mitochondrial dysfunction by regulating VDAC1, thereby reducing MI/RI injury. This provides a novel potential target for the prevention and treatment of ischaemic cardiomyopathy.

## 2 Materials and Methods

### 2.1 Materials and Chemical Agents

Ferrostatin‐1(Fer‐1, ferroptosis suppressor) was purchased from MCE Bioreagents. The adenoviral vectors pAD/VDAC1 or pAD/VDAC1‐Negative Control (NC) were acquired from Genepharma Co. Ltd. The adenoviral vectors pAD/FTMT or pAD/FTMT‐Negative Control (NC) were acquired from Genechem Co. Ltd. RiboBio constructed si‐VDAC1. The antibodies to PTGS2, GPX4 and Horseradish peroxide‐coupled IgG were procured from Zen‐bio; VDAC1 was bought from Proteintech; and FTMT was obtained from MyBioSource. Antibodies against β‐actin were acquired from ZSGB‐BIO.

### 2.2 H9c2 Cells Culture, Adenovirus/Si‐RNA Transduction and Anoxia/Reoxygenation (A/R) Constructs

The H9c2 line was obtained from the National Collection of Authenticated Cell Cultures (https://www.cellbank.org.cn/) and cultivated in high‐glucose Dulbecco's modified Eagle's medium (H‐DMEM, HyClone) adding 10% premium plus, heat inactivated foetal bovine serum (FBS) and 1% penicillin–streptomycin‐glutamine (PSG; 100×) (GIBCO) at 37.0°C, 21.0% O_2_ and 5.0% CO_2_, 95.0% humidity. H9c2 cells were maintained with medium and pre‐incubated with pAD/VDAC1, pAD/FTMT or NC, and si‐VDAC1or NC for 48 h [18]. The validation results are shown in Figure S1A,B and Figure S2A,B. The sequence of rat VDAC1 gene [NCBI (National Center for Biotechnology Information) reference sequence NM_031353.1], rat FTMT gene [NCBI reference sequence NM_001106136] and si‐VDAC1 are shown in Table S1.

As in previous studies [19], the A/R model was employed to simulate in vivo MI/RI conditions. In brief, cells were exposed to anoxic medium and a concentration of 95.0% N_2_ and 5.0% CO_2_ for 4 h at 37.0°C for anoxia. Following that, cells were incubated in reoxygenation liquid under a humidified chamber at 37.0°C, 95.0% O_2_ and 5.0% CO_2_ for 3 h, using it to simulate MI/RI, and the following experiments were performed.

### 2.3 Experiment Designing

The H9c2 cells are randomly designed in the following way: (a) Control group; (b) A/R group: cardiomyocytes were incubated in A/R medium; (c) pAD/VDAC1+ A/R group: cardiomyocytes were pretreated with pAD/VDAC1 for 48 h before being exposed to A/R injury; (d) pAD/VDAC1+ pAD/FTMT+ A/R group: A/R pre‐injury, cardiomyocytes pre‐incubated with pAD/VDAC1+ pAD/FTMT 48 h; (e) pAD/FTMT+ A/R group: pAD/FTMT incubated for 48 h prior to A/R; (f) pAD/VDAC1‐NC + pAD/FTMT‐NC+ A/R (NC + AR) group: A/R Pre‐injury, cardiomyocytes pre‐treated with pAD/VDAC1‐NC and co‐treated with pAD/FTMT‐NC for 48 h; (g) Fer‐1+ A/R group: cardiomyocytes pre‐treated with 10 μM Fer‐1 [19] for 2 h and then exposed to A/R for 4/3 h.

### 2.4 Cell Viability and Lactate Dehydrogenase (LDH) Assessment

The viability of cells was evaluated utilising the cell counting kit‐8 (CCK‐8) assay (GIBCO, catalogue# no. GK10001) in accordance with the product's guidelines [28].

Culture supernatants were retained following A/R‐induced LDH release levels. LDH release levels were measured utilising an LDH assay kit (Beyotime, Shanghai, China, catalogue# no. C0017) following the product's instructions [29].

### 2.5 Evaluation of Cellular Iron, Malondialdehyde (MDA), Superoxide Dismutase (SOD) and Glutathione/Glutathione Disulfide (GSH/GSSG)

Following A/R‐treated, the release levers of cellular MDA, SOD, GSH/GSSG, GPX and total iron ion form cells lysate supernatant were quantified utilising MDA assay kit (Shanghai, China, Beyotime, catalogue# no. S0131M), SOD assay kit (Shanghai, China, Beyotime, catalogue# no. S0101M), GSH and GSSG assay kit (Shanghai, China, Beyotime, catalogue# no. S0053) and total iron ion colorimetric assay kit (APPLYGEN, catalogue# no. E1042‐100), respectively, following the product's guidelines [19].

### 2.6 Measurement of Intracellular ROS and Lipid ROS

A DCFH‐DA kit (Shanghai, China, Beyotime, catalogue# no. S0033S) was used to measure intracellular ROS content, following the manufacturer's instructions [18]. In brief, H9c2 cells were A/R‐treated and then stained with 1 mL no‐FBS medium containing 1 μL DCFH‐DA and 10 μL Hoechst 33342 (Shanghai, China, Beyotime, catalogue# no. C1027) at 37.0°C for 25 min in the darkness. The level of LIP ROS was evaluated using the C^11^‐BODIPY581/591 kit (GlpBio, US, catalogue# no. GC40165). Briefly, cells were stained with 10 μM C^11^‐BODIPY581/591 and 10 μL/mL Hoechst 33342 (Shanghai, China, Beyotime, catalogue# no. C1027) at 37.0°C for 35 min in the absence of light. The intracellular ROS and LIP ROS levels in cells were measured using an Olympus IX 73 microscope (Tokyo, Japan, Olympus).

### 2.7 Determination of Intracellular Ferrous Iron Content

Intracellular levels of ferrous iron were quantified using the FerroOrange kit (Tokyo, Japan, DOJINDO, catalogue# no. F374) following the manufacturer's instructions [30]. Briefly, after A/Rtreatment, H9c2 myofibroblasts were incubated with 1 μM FerroOrange at 37°C for 30 min while away from light. Excess FerroOrange was removed by rinsing once with HBSS, and the ferrous iron content was assessed under an Olympus fluorescence microscope (Tokyo, Japan).

### 2.8 Western Blotting

The cell samples were hydrolysed in western and IP cell lysates (Beyotime, catalogue# no. P0013) with a 1% Protease Inhibitor Cocktail (GLPBIO, US, catalogue# no. GK10014). BCA assay kit (Beyotime, catalogue# no. P0012) for protein concentration quantification. The proteins were renatured by adding 1x sodium dodecyl sulphate‐polyacrylamide gel electrophoresis (SDS‐PAGE) protein buffer (Beyotime, catalogue# no. P0015F) and boiling at 99.9°C for 10 min in a metal heater. Then, a 20‐40 μg protein sample was added to each lane for separation using 10% PAGE gel fast preparation kit (Epizyme, catalogue# no. PG112), and then transferred to a polyvinylidene fluoride membrane that was blocked with 5% non‐fat dry milk for 2 h in a three‐buffer brine containing 0.10% Tween‐20. The membrane was then incubated overnight in a low‐speed shaker maintained at 4°C with primary antibodies against VDAC1 (1:1000; Proteintech, catalogue# no. 55259‐1‐AP), FTMT (1:1000; MyBioSource, catalogue# no. MBS2002952), PTGS2 (1:800; ZENBIO, catalogue# no. R23969), GPX4 (1:800; ZENBIO, no. 381958) and β‐actin (1:2000; ZSGB‐BIO, catalogue# no. TA‐09). The next day, after completing the membrane washing procedure, the membrane was incubated with a secondary Goat Anti‐Rabbit/Mouse IgG H&L‐conjugated antibody (1:5000; ZENBIO, catalogue# no. 511203; catalogue# no. 511103) for 2 h at room temperature. The membrane was washed to remove excess antibodies and then visualised using the BeyoECL Plus kit (Beyotime, catalogue# no. P0018S). The intensity of grey values in the protein lanes was measured using the ImageJ software (National Institutes of Health).

### 2.9 Flow Cytometry Analysis

mitochondrial permeability transition pore (MPTP) opening and mitochondrial membrane potential (MMP) were assessed utilising the MPTP assay kit (BESTBIO, catalogue# no. BB‐48122) and MMP assay kit (BESTBIO, catalogue# no. BB‐4105), respectively, adhering to the manufacturer's instructions [19]. To detect MPTP, H9c2 cardiomyocytes were stained with BBcellProbe M61 working solution and quencher, followed by incubation at 37.0°C for 15 min, avoiding exposure to light, then removal of the supernatant, and cell resuspension in 600 μL HBSS. To assess MMP, H9c2 cells were incubated with the JC‐1 probes, followed by incubation at 37°C for 20 min protected from light, washing twice with PBS and resuspending the cells in 600 μL PBS. For apoptosis, in brief, 1 × 10^6^ cells/tube were collected after A/R‐induced and placed in 600 μL of 1X Annexin incubating solution, staining with 5 μL Annexin V‐FITC and 10 μL PI (BESTBIO, catalogue# no. BB‐4101) in succession and incubated in the dark for 15 min and 5 min at 6°C, respectively. The levels of LIP ROS, MPTP and MMP from cells were then immediately analysed using Agilent NovoCyte Advanteon flow cytometer [Annexin V‐FITC/PI: excitation (EX) at 488 nm, emission (EM) at 580 nm; MPTP: EX at 488 nm, EM at 520 nm; MMP: EX at 485 nm, EM at 580 nm]. Flow cytometry data were analysed with the FlowJo software.

### 2.10 Animal Experiments

The adult male Sprague–Dawley (SD) rats, 200 ± 20 g, were acquired from the Animal Center of Nanchang University (Nanchang, China). The experimental procedures followed the guidelines of the ARRIVE and the National Institutes of Health (NIH) guidelines, and were authorised by the Animal Experiment Ethics Committee of the First Affiliated Hospital of Nanchang University (Approval No. CDYFY‐IACUC‐202312QR010). Following 1 week of acclimatisation feeding, 40 SD rats were systematically randomised into five groups (*n* = 8 per group):(1) Sham group, (2) MI/RI group, (3) MI/RI + pAD/VDAC1 group, (4) MI/RI + pAD/VDAC1 + pAD/FTMT group and (5) MI/RI + pAD/FTMT group. The rats in the MI/RI + pAD/VDAC1 group were injected intracardially with pAD/VDAC1 (~2 × 10^11^ plaque‐forming units/ml distributed in 3–5 sites, approximately 25 μL); rats in the MI/RI + pAD/FTMT group were administered with an intramyocardial injection of pAD/FTMT (~2 × 10^11^ plaque‐forming units/ml, distributed across 3–5 sites about 25 μL); rats belonging to the MI/RI + pAD/VDAC1 + pAD/FTMT group underwent the same pAD/VDAC1and pAD/FTMT treatment regimen as the MI/RI + pAD/VDAC1 group and MI/RI + pAD/FTMT group. Five days later, based on the results of in vivo validation (Figure S3A,B), the MI/RI model was constructed by ligating the left coronary artery of the anterior descending branch (LAD) [29, 31]. Before surgery, rats were anaesthesia‐induced with 3% isoflurane. Subsequently, the rats were placed supine on the operating table, connected to a small animal ventilator, and a left 3–4 intercostal incision was made to expose the heart. The LAD was then ligated with a 7–0 silk suture for 45 min to induce myocardial ischaemia, followed by suture release to allow for 24 h of reperfusion. Conversely, the Sham surgical group underwent the same surgical protocol without actually clamping the LAD.

### 2.11 Echocardiography

Following MI/RI, rats were anaesthetised with 3% isoflurane and then cardiac function, including left ventricular ejection fraction (LVEF), left ventricular shortening fraction (LVFS), left ventricular internal dimension‐diastole (LVIDd), left ventricular internal diameter in systole (LVIDs) and heart rate (HR) were assessed using 2D transthoracic echocardiography based on the Vevo 2100 imaging system [32] (VisualSonics, Canada).

### 2.12 Assessment of CK‐MB, LDH, Iron Contents and MDA

After ultrasound assessment, rats in each group were euthanised using a combination of anaesthesia (anaesthesia‐induced with 3% isoflurane) followed by cervical dislocation, and serum and heart samples were rapidly collected. Subsequently, the contents of CK‐MB, LDH and Fe^2+^ in serum and total iron and MDA in myocardial tissues were assayed using the CK‐MB, LDH and Fe^2+^ assay kit (Nanjing Jianjian, Nanjing, China) and the MDA kit (Biyuntian, Shanghai, China), respectively, according to the manufacturer's instructions [33].

### 2.13 Measurement of the Area at Risk

The infarct area was assessed by double staining with Evans blue and triphenyl tetrazolium chloride (TTC) [34]. After reperfusion, the LAD was clamped, and subsequently, 0.5% Evans blue dye was injected into the left ventricle. Hearts were rapidly excised, snap‐frozen in liquid nitrogen for 1 min and cut into 5 equal transverse sections. These slices were then incubated in 2% TTC (Solarbio, Beijing, China) for 20 min at room temperature. After incubation, the heart sections were fixed in paraformaldehyde (PFA) for 24 h, and images were acquired for analysis of the area at risk = the red (ischaemic but non‐infarcted) + greyish‐white (infarcted) areas.

### 2.14 HE Staining

Briefly, rat hearts were fixed with 4% PFA, dehydrated with ethanol, embedded in paraffin and sectioned. The sections were then incubated with HE dye according to the instructions [35], and the sections were observed with a light microscope (Olympus, Tokyo, Japan).

### 2.15 DHE Staining

Following MI/RI treatment, heart samples were collected, dehydrated, frozen, embedded and sectioned, and then stained with dihydroethidium (DHE) probe (Yessen, China) for 1 h at 37°C according to the manufacturer's instructions [36]. Finally, images were acquired using a fluorescence microscope (Nikon Eclipse Ci, Japan).

### 2.16 Evaluation of Mitochondrial Ultrastructure Using Transmission Electron Microscopy (TEM)

Following A/R(MI/RI)‐treated, H9c2 myofibroblasts (cardiac tissue) were incubated with glutaraldehyde overnight. The specimens were then fixed, dehydrated, washed, embedded and ultrathin‐sectioned (~50 nm) before being stained. The mitochondrial ultrastructure was assessed using TEM (Hitachi 7800), and the degree of damage was evaluated using the Flameng score method [37].

### 2.17 Statistical Analysis

Statistical analyses were statistically analysed using GraphPad Prism 9.0; the data were performed as mean ± standard deviation (SD). *p* ≤ 0.05 was regarded as statistical significance.

## 3 Results

### 3.1 Activation of VDAC1 Exacerbates A/R‐Associated Injury and Oxidative Stress

**FIGURE 1:**
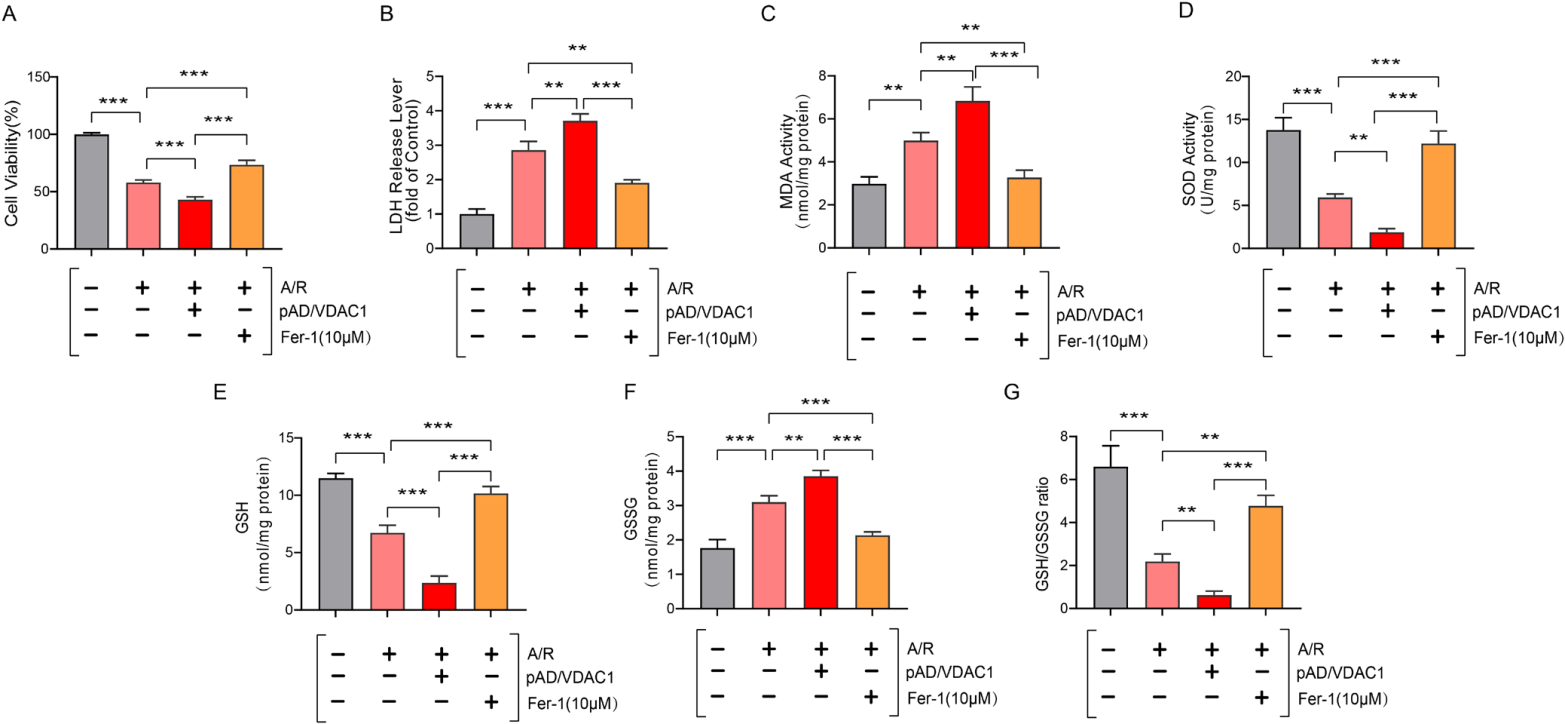
Activation of VDAC1 exacerbates A/R‐associated injury. (A) CCK‐8 assessment of A/R‐induced cell viability after pAD/VDAC1, Fer‐1 pre‐treatment. (B) LDH, (C) MDA, (D) SOD, (E) GSH, (F) GSSG and (G) GSH/GSSG ratio. Data are presented as the mean ± SD (*n* = 3), **p < 0.01, ***p < 0.001.

The specific molecular mechanisms of VDAC1 action in A/R‐induced cardiomyocytes were explored through the testing of a variety of functional indicators and enzymology, which were subjected to different experiments. In our results, cell survival and LDH content were significantly reduced/elevated in the A/R group, compared with the control group (Figure 1A,B). This result indicated that the A/R model was successfully constructed. Conversely, LDH levels were markedly diminished and cell activity was substantially enhanced in the Fer‐1 + A/R group. Similarly, the activity of SOD, the content of GSH and the ratio of GSH to GSSG, which are indicators of oxidative stress, were significantly reduced, and the levels of MDA and GSSG were significantly elevated following A/R induction. These effects were reversed by pretreatment with Fer‐1, a ferroptosis inhibitor. However, the expression of VDAC1 was activated using pAD/VDAC1 pretreatment, which resulted in a further promotion of A/R injury (Figure 1C–G). The results demonstrate that the activation of VDAC1 may exacerbate A/R‐induced oxidative stress injury. Furthermore, si‐VDAC1 pretreatment has been shown to effectively ameliorate A/R‐associated oxidative stress injury (Figure S2C–E). However, it remains unclear whether the cardiac damaging effects of VDAC1 activation are associated with ferroptosis.

**FIGURE 2:**
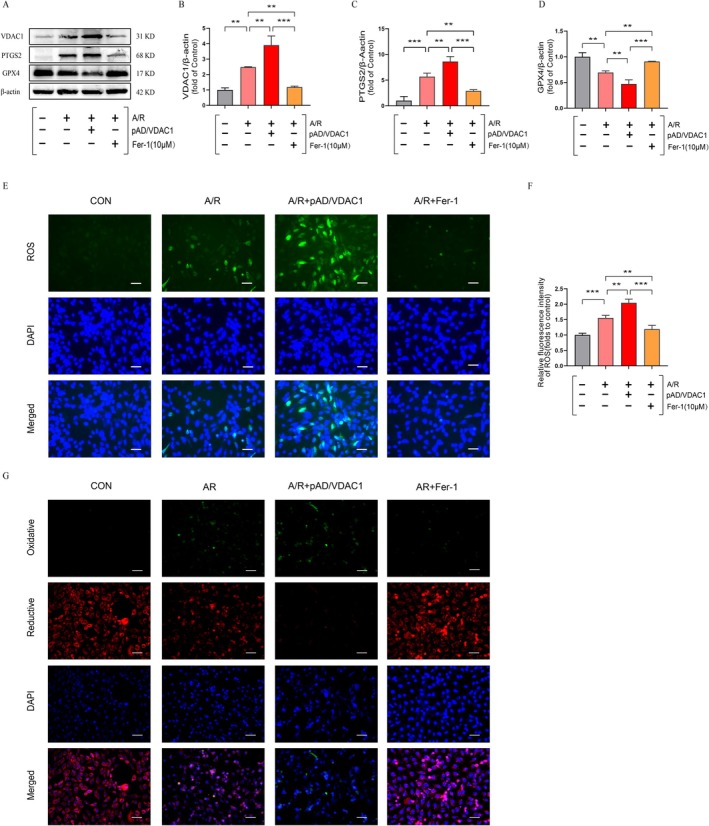
Activation of VDAC1 increases A/R‐associated ferroptosis sensitivity. (A–D) Protein expression of VDAC1, PTGS2 and GPX4 was determined using Western blot analysis in cell lysates after A/R‐induced following pre‐treatment with pAD/VDAC1, Fer‐1. (E, F) ROS levels were quantified by immunofluorescence (enlargement, ×200; scaled bar: 100 μm). (G) lipid ROS (enlargement, ×200; scaled bar: 100 μm). Data are presented as the mean ± SD (*n* = 3), **p < 0.01, ***p < 0.001.

### 3.2 Activation of VDAC1 Increases A/R‐Associated Ferroptosis Sensitivity

To explore the relationship between VDAC1 and ferroptosis in A/R. We examined ferroptosis‐related indicators. PTGS2/GPX4 serve as positive/negative markers of ferroptosis, respectively. Our results demonstrate a remarkable elevation in the protein expression level of PTGS2 and a significant decline in GPX4 in the A/R group relative to the control group (Figures 2A,C,D). This aligns with the findings of previous studies. In addition, the protein expression of VDAC1 was markedly elevated following injury in the A/R group (Figures 2A,B). Conversely, the protein expression levels of PTGS2 and GPX4 were markedly diminished or enhanced following Fer‐1 pretreatment. However, pretreatment with pAD/VDAC1 significantly activated VDAC1 expression, while GPX4 protein expression was significantly inhibited and PTGS2 was significantly elevated (Figures 2A–D). ROS has been identified as an important factor in the induction of ferroptosis. The levels of ROS and lipid ROS were quantified by immunofluorescence. Similarly, the levels of ROS and lipid ROS were significantly elevated after A/R induction. The activation of VDAC1 expression using pAD/VDAC1 pretreatment further promoted ROS and lipid ROS bursts. The A/R‐associated effect was reversed by Fer‐1 pretreatment (Figures 2E–G). The findings indicate that VDAC1 plays a role in the A/R‐induced ferroptosis process and that the activation of VDAC1 enhances A/R‐associated ferroptosis sensitivity.

### 3.3 Overexpression of VDAC1 Promotes A/R‐Associated Ferroptosis Through Downregulation of FTMT

**FIGURE 3:**
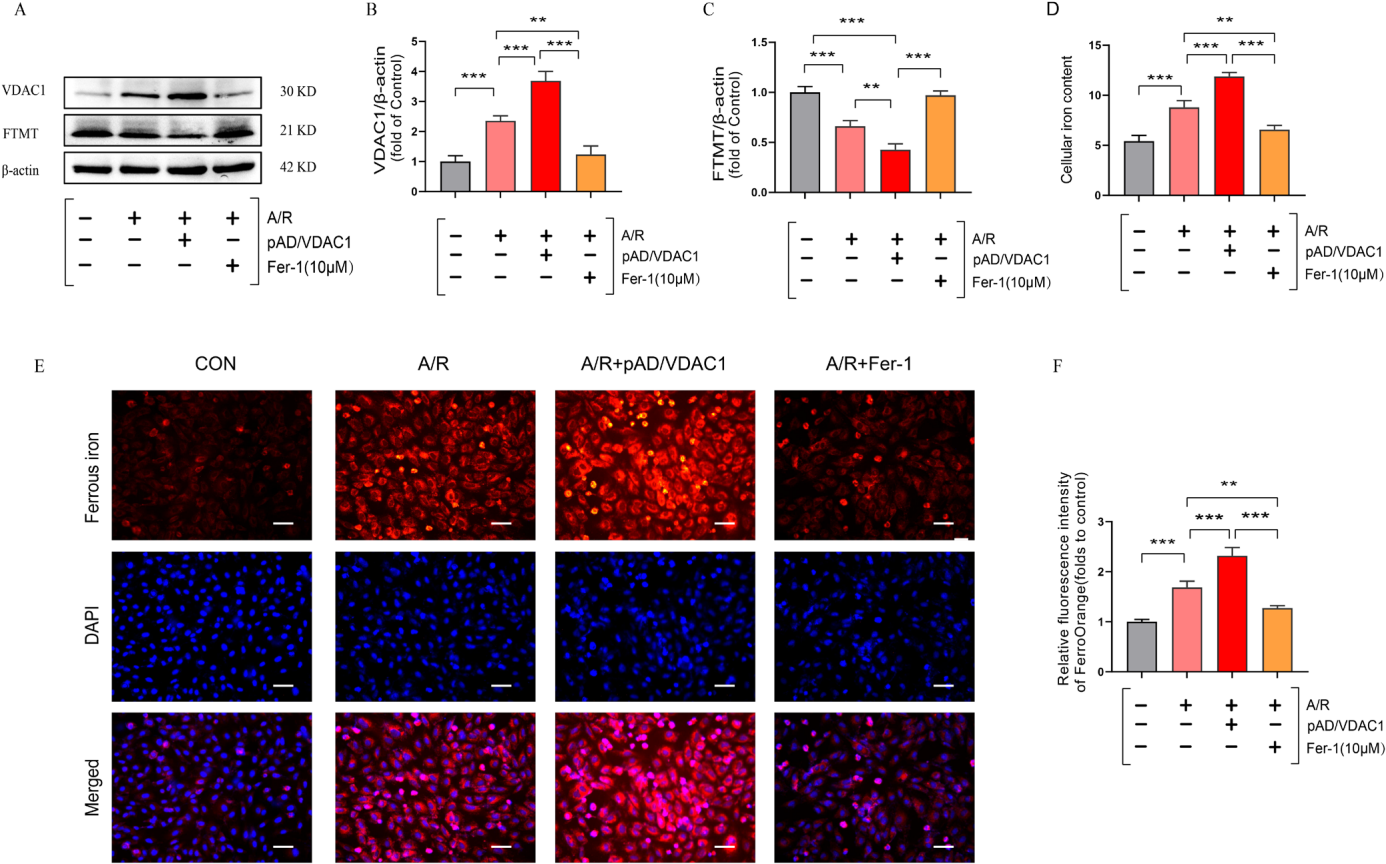
Overexpression of VDAC1 promotes A/R‐associated ferroptosis through downregulation of FTMT. (A–C) Protein expression of VDAC1 and FTMT was determined using Western blot analysis in cell lysates after A/R‐induced following pre‐treatment with pAD/VDAC1, Fer‐1. (D) Total iron conten. (E, F) FerroOrange probe for detecting ferrous ions (enlargement, 200×; scaled bar: 100 μm). Data are presented as the mean ± SD (*n* = 3). **p < 0.01, ***p < 0.001.

Ferroptosis is a recently discovered new form of regulatory cell death characterised by iron overload. Therefore, cellular iron and free ferrous ions were examined. The findings demonstrated that cellular iron levels were markedly elevated in the A/R group relative to the control group, whereas Fer‐1 pretreatment resulted in a notable reduction in cellular iron levels. The activation of VDAC1 using pAD/VDAC1 pretreatment further aggravated cellular iron deposition (Figure 3D). Similarly, the detection of intracellular free ferrous iron content using the FerroOrange probe demonstrated that Fer‐1 effectively attenuated A/R‐induced iron overload, whereas pAD/VDAC1 pretreatment resulted in a further exacerbation of free ferrous iron accumulation (Figure 3E,F). It is noteworthy that the expression level of FTMT protein was found to be reduced, while that of VDAC1 was elevated in the A/R group in comparison to the control group. Furthermore, the activation of VDAC1 expression was observed to result in further suppression of FTMT protein expression. Conversely, Fer‐1 was observed to reverse the favourable alterations (Figure 3A–C). In addition, we silenced VDAC1 expression by si‐VDAC1 treatment and showed that knockdown of VDAC1 effectively attenuated A/R‐induced ferroptosis‐related injury indexes compared with the A/R group (Figure S2F–K). These findings indicate VDAC1 plays a critical role in the A/R‐induced ferroptosis process and that overexpression of VDAC1 may contribute to A/R‐associated ferroptosis through the downregulation of FTMT.

### 3.4 Overexpression of FTMT Alleviates A/R‐Induced Injury and Oxidative Stress by Inhibiting VDAC1 Activation

**FIGURE 4:**
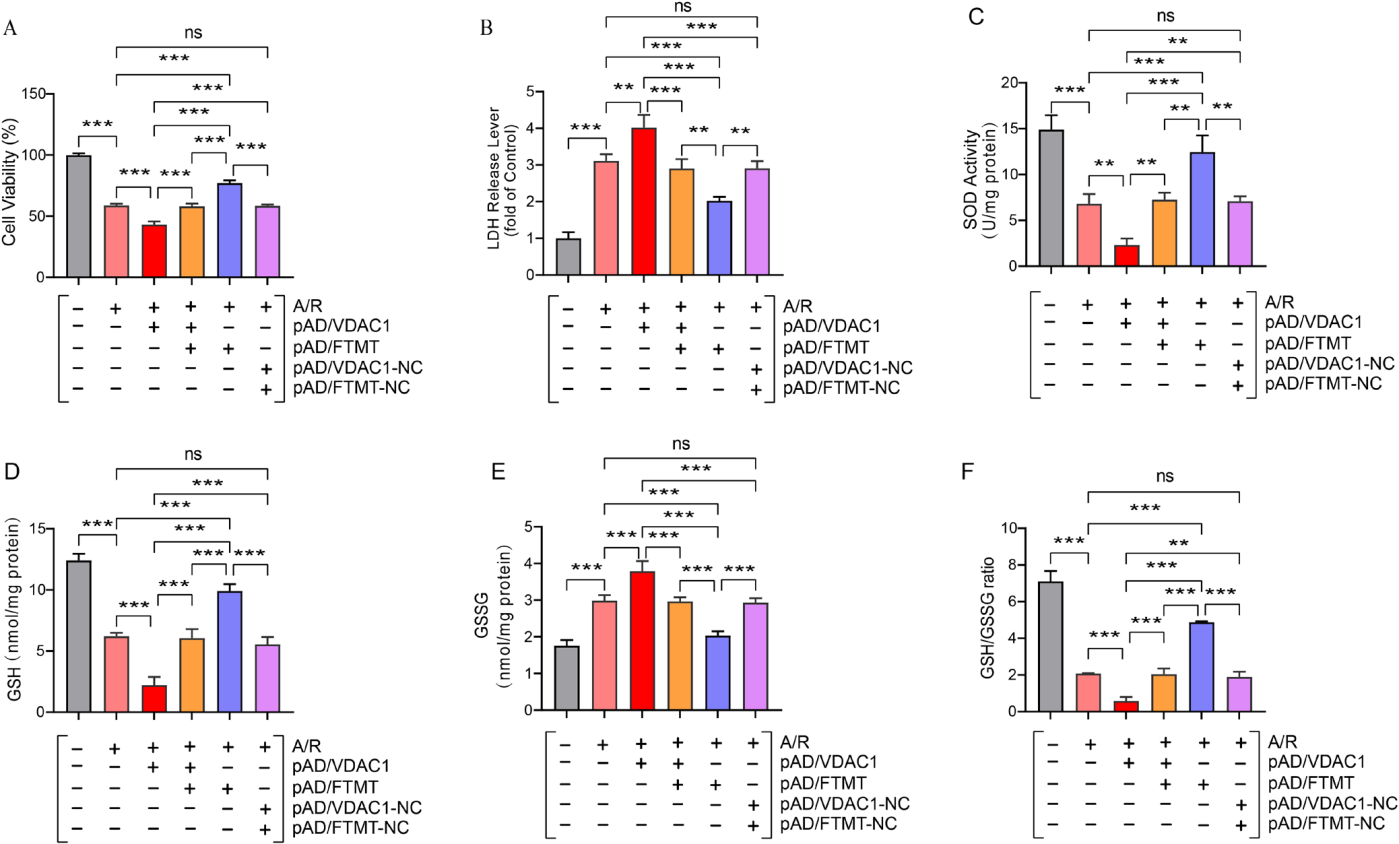
Overexpression of FTMT alleviates A/R‐induced injury and oxidative stress by inhibiting VDAC1 activation. (A) CCK‐8 assay of A/R‐induced H9c2 cardiomyocyte viability after pAD/VDAC1, pAD/FTMT or NC pre‐treated. (B) LDH, (C) MDA, (D) SOD, (E) GSH, (F) GSSG and (G) GSH/GSSG ratio. Data are presented as the mean ± SD (*n* = 3). **p < 0.01, ***p < 0.001; ns, not significant.

Further research is required to elucidate the precise mechanism of VDAC1/FTMT and iron pigmentation in A/R‐related processes. Subsequently, the impact of FTMT overexpression on A/R and its correlation with VDAC1 was evaluated. The results demonstrated that cell survival/LDH was significantly decreased/increased by exposure to A/R. In the pAD/FTMT+ A/R group, overexpression of FTMT was observed to significantly reduce LDH levels and increase cell survival, compared with the A/R group. Furthermore, in the pAD/FTMT+ pAD/VDAC1+ A/R group, overexpression of FTMT was observed to significantly inhibit the A/R injury promoted by pAD/VDAC1 preconditioning, compared with the pAD/VDAC1+ A/R group (Figure 4A,B). Similarly, the levels of SOD, GSH and the GSH/GSSG ratio were found to be decreased, while the level of GSSG was increased in the A/R group when compared with the control group. pAD/VDAC1 pretreatment aggravated A/R‐induced injury. The oxidative stress damaging effect of activated VDAC1 on the myocardium was mitigated by pAD/FTMT (Figure 4C–F). Our findings indicate that overexpression of FTMT attenuates A/R‐induced injury and oxidative stress by inhibiting VDAC1 activation.

### 3.5 Overexpression of FTMT Inhibits A/R‐Induced Ferroptosis by Hindering VDAC1 Activation

**FIGURE 5:**
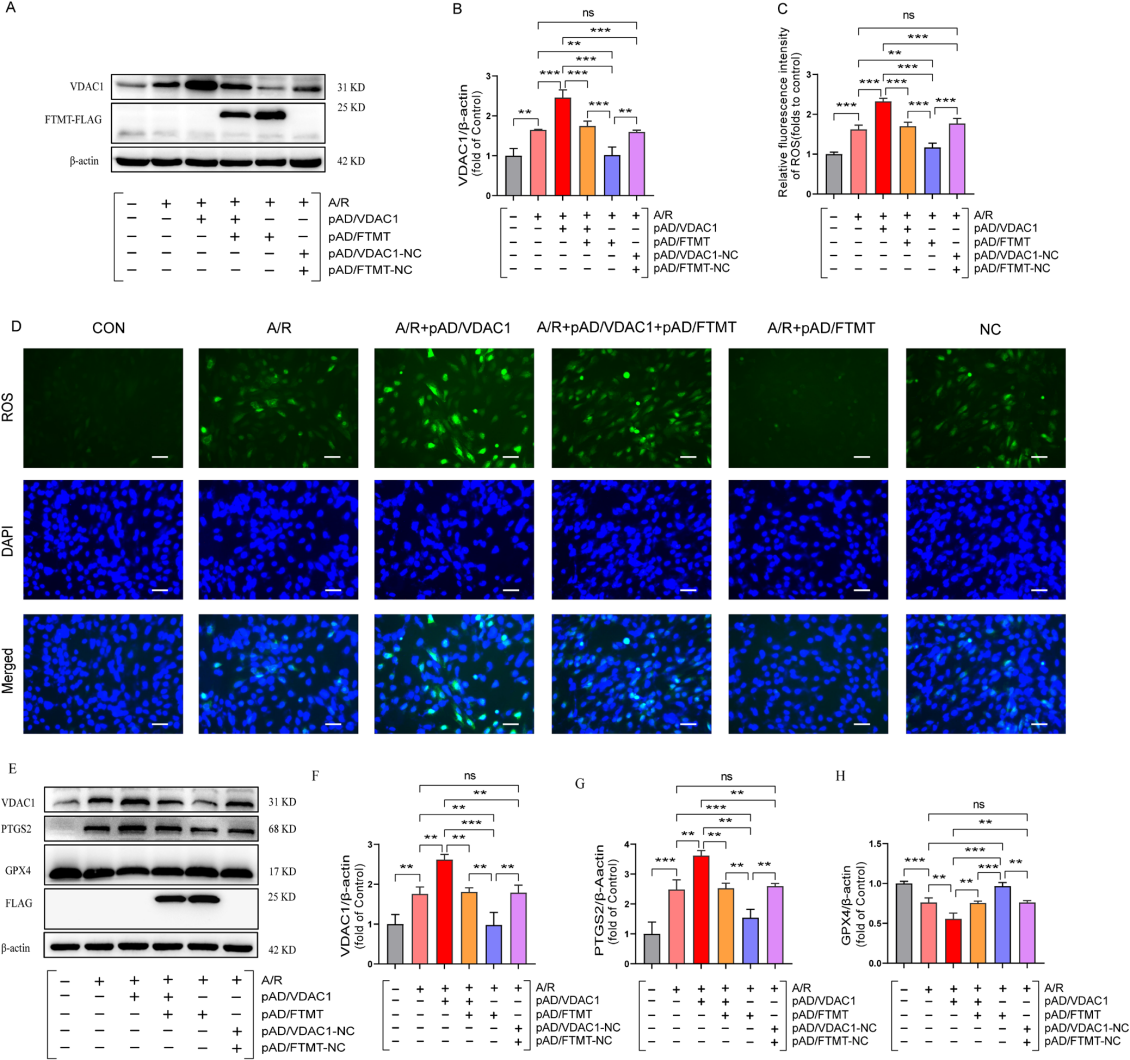
Overexpression of FTMT inhibits A/R‐induced ferroptosis by hindering VDAC1 activation. (A, B) Expression of VDAC1 and FTMT‐FLAG proteins in A/R‐induced cells were determined using western blot analysis after pre‐treatment with pAD/VDAC1, pAD/FTMT or NC. (C, D) ROS levels were quantified by immunofluorescence (enlargement, ×200; scaled bar: 100 μm). (E–H) Western blot analysis for detecting ferroptosis‐related protein expression in cell lysates after treatment. Data are presented as the mean ± SD (*n* = 3). **p < 0.01, ***p < 0.001; ns, not significant.

**FIGURE 6:**
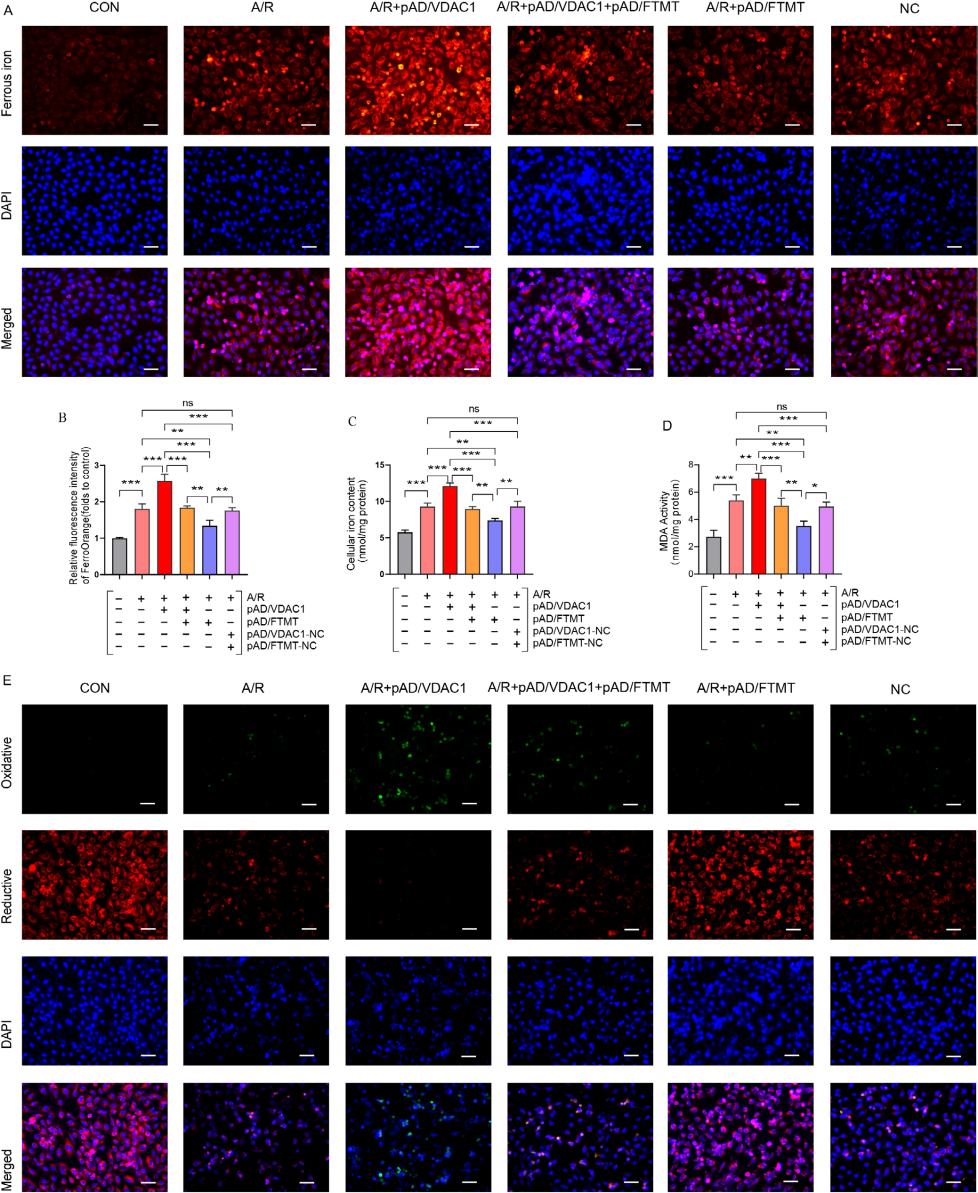
Overexpression of FTMT ameliorates A/R‐induced accumulation of iron and lipid ROS by adjusting VDAC1. (A, B) Immunofluorescence for detecting ferrous ions following pre‐treatment with pAD/VDAC1, pAD/FTMT or NC (enlargement, 200×; scaled bar: 100 μm). (C) total iron content. (D) MDA. (E) Fluorescent probe C^11^‐BODIPY581/591 for detecting lipid ROS (enlargement, ×200; scaled bar: 100 μm). Data are presented as the mean ± SD (*n* = 3). *p < 0.05, **p < 0.01, ***p < 0.001, ns, not significant.

To further elucidate whether overexpression of FTMT inhibits A/R‐induced ferroptosis by hindering VDAC1 activation, the association between FTMT and VDAC1 at the protein molecular level was first assessed by western blotting. The results demonstrated that A/R exposure led to the activation of VDAC1. Furthermore, the activation of VDAC1 was more pronounced in the presence of pAD/VDAC1 pretreatment. Conversely, in the pAD/FTMT+ pAD/VDAC1+ A/R group, overexpression of FTMT markedly impeded VDAC1 activation compared with the pAD/VDAC1+ A/R group (Figures 5A,B). Additionally, in the pAD/FTMT+ A/R group, overexpression of FTMT significantly inhibited A/R‐induced VDAC1 activation in comparison to the A/R group. Subsequently, the protein expression levels of GPX4 and PTGS2 were evaluated. As illustrated in (Figures 5E–H), the activation of VDAC1 was found to markedly reduce the protein expression of GPX4 while elevating the PTGS2 protein level. However, the overexpression of FTMT effectively counteracted these effects associated with pAD/VDAC1 pretreatment. Furthermore, we examined ROS, lipid ROS, MDA and iron ions. The results demonstrated a significant elevation in ROS, lipid ROS, MDA and free iron levels following AR injury, in comparison to the control group. In the pAD/VDAC1+ A/R group, activation of VDAC1 further promoted the accumulation of ROS, lipid ROS, MDA, and free iron. In contrast, pAD/FTMT pretreatment significantly eliminated the deleterious effects of VDAC1 activation (Figures 5C,D) (Figures 6A–E). These results confirm that overexpression of FTMT inhibits A/R‐induced ferroptosis by hindering VDAC1 activation.

### 3.6 Overexpression of FTMT Ameliorates A/R‐Induced Mitochondrial Dysfunction by Mediating VDAC1

**FIGURE 7:**
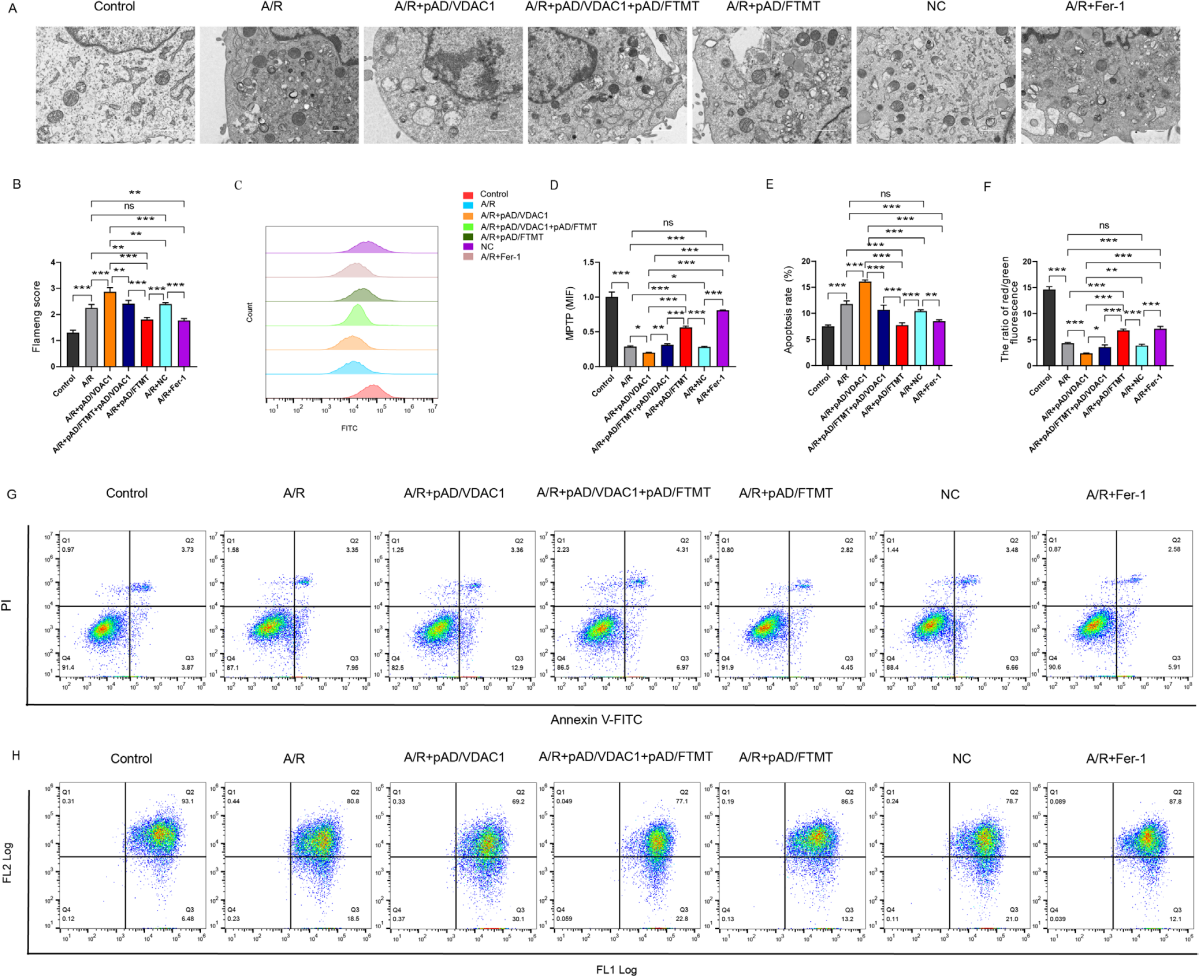
Overexpression of FTMT ameliorates A/R‐induced mitochondrial dysfunction by mediating VDAC1. (A, B) TEM for assessing mitochondrial ultrastructure (enlargement, 8000×; scaled bar: 1 μm). (C, D) Flow cytometry BBcellProbe M61 assay for cellular MPTP. (E, G) Annexin V‐FITC/PI assay for apoptosis. (F, H) JC‐1 assay for cellular MMP. Data are presented as the mean ± SD (*n* = 3). *p < 0.05, **p < 0.01, ***p < 0.001, ns, not significant.

Prior research has demonstrated that mitochondria are among the most crucial organelles in cardiomyocytes, and that ferroptosis is typified by major pronounced morphological alterations in mitochondria. Accordingly, an assessment of the alterations in mitochondrial ultrastructure was conducted using TEM (Figure 7A,B). The results demonstrated that following exposure to A/R injury, mitochondrial morphology was markedly altered, exhibiting a reduction in volume, a loss of ridges, and a considerable elevation in Flameng scores. pAD/VDAC1 preconditioning activated VDAC1, which further exacerbated mitochondrial injury. In contrast, overexpression of FTMT significantly blocked VDAC1 activation, thereby attenuating A/R‐induced mitochondrial injury. Fer‐1 pretreatment effectively alleviated mitochondrial injury. This indicates that FTMT exerts a Fer‐1‐like effect. In addition, we evaluated the degree of MPTP opening, apoptosis and MMP levels in mitochondria (Figure 7C–H). The results demonstrated that MPTP was significantly opened, apoptosis was increased considerably, and MMP was significantly reduced in the A/R group in comparison to the control group. pAD/VDAC1 pretreatment activated VDAC1, which further promoted MPTP over‐opening and MMP reduction. However, these changes were significantly inhibited by pAD/FTMT and Fer‐1 pretreatment. Our results suggest that overexpression of FTMT ameliorates A/R‐induced mitochondrial dysfunction by mediating VDAC1.

### 3.7 Overexpression of FTMT Alleviates MI/RI‐Induced Myocardial Injury by Mediating VDAC1

**FIGURE 8:**
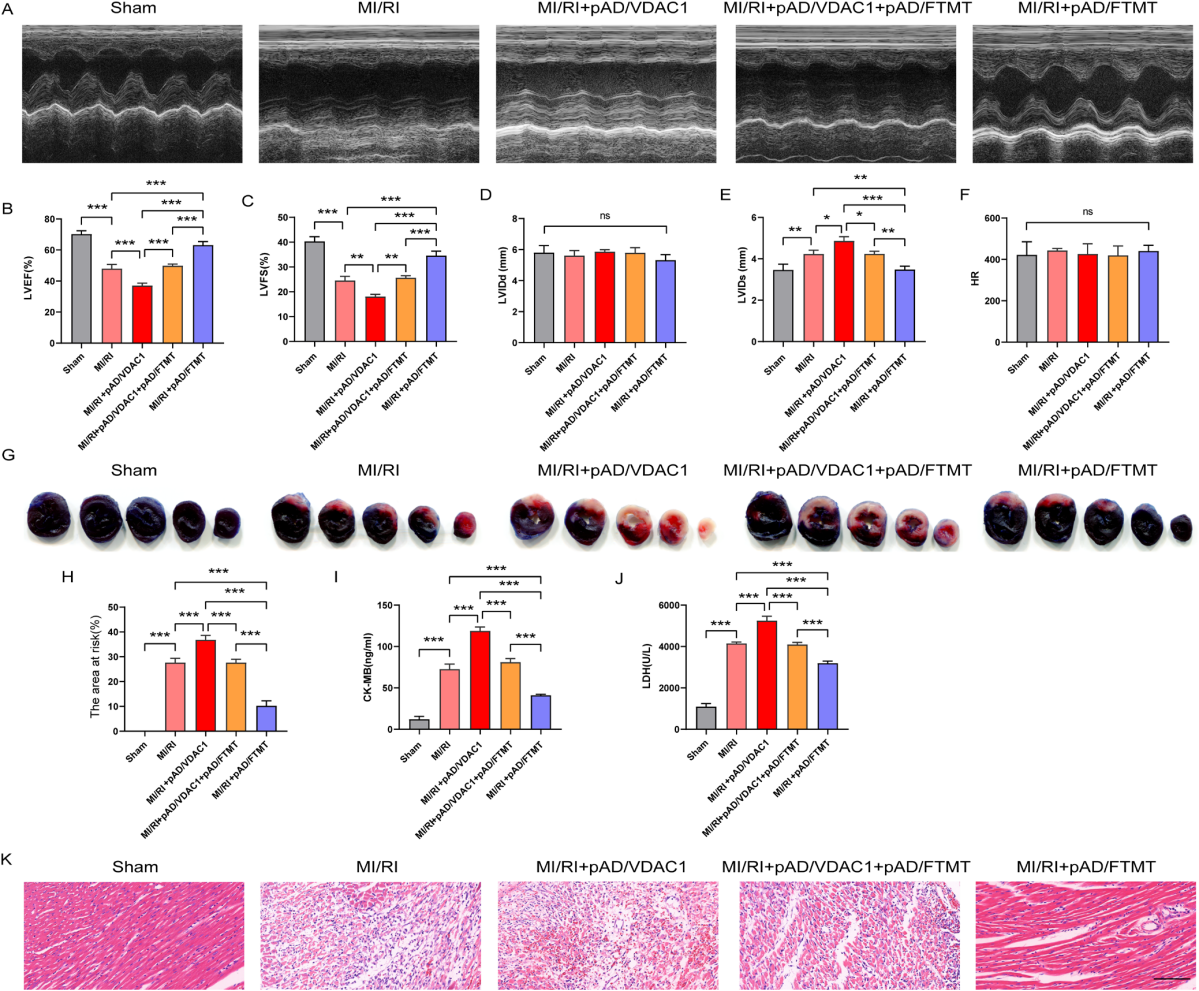
Overexpression of FTMT alleviates MI/RI‐induced myocardial injury by mediating VDAC1. (A) Representative images of rat heart detected by echocardiography. (B) LVEF. (C) LVFS. (D) LVIDd. (E) LVIDs. (F) HR. (G, H) Quantitative data and representative images of the area at risk after MI/RI‐induced. (I) CK‐MB levels in serum of rats in each group. (J) LDH. (K) Representative images of rat myocardial tissue damage by HE staining (enlargement, 200×, scaled bar: 100 μm). Data are presented as the mean ± SD (*n* = 3), **p < 0.01, ***p < 0.001.

To assess the role of VDAC1 and FTMT in MI/RI, we constructed an MI/RI model using SD rats. The results of cardiac echocardiography showed that in the MI/RI injury group, cardiac function was significantly impaired, as evidenced by a significant reduction in left ventricular ejection fraction (LVEF) and left ventricular shortening fraction (LVFS). Moreover, cardiac function impairment was further aggravated after pAD/VDAC1 intervention. Similarly, a consistent trend of elevated levels of cardiac enzyme injury biomarkers (CK‐MB and LDH) was observed in the serum of rats in the MI/RI group and the MI/RI + pAD/VDAC1 group. In contrast, overexpression of FTMT partially alleviated the serological biomarkers and cardiac function impairment induced by pAD/VDAC1 intervention. Notably, pAD/FTMT single transduction effectively ameliorated the aforementioned manifestations of impairment in MI/RI (Figure 8A–F,I,J). Similarly, the extent of myocardial infarction was significantly greater in the MI/RI group than in the Sham group, and pAD/VDAC1 intervention promoted myocardial infarction. Importantly, overexpression of FTMT blocked the pAD/VDAC1 damaging effect and significantly reduced the infarct size (Figure 8G,H). In addition, HE pathology sections showed significant damage to myocardial fibres and disorganisation of cardiomyocytes in the MI/RI group. Similarly, pre‐injection of pAD/VDAC1 intracardially further exacerbated the MI/RI‐induced histopathological and morphological changes in myocardial tissue. In contrast, overexpression of FTMT reversed the detrimental effects of pAD/VDAC1 on MI/RI in vivo and effectively ameliorated MI/RI injury (Figure 8K).

### 3.8 Overexpression of FTMT Inhibits MI/RI‐Induced Ferroptosis and Mitochondrial Injury by Mediating VDAC1

**FIGURE 9:**
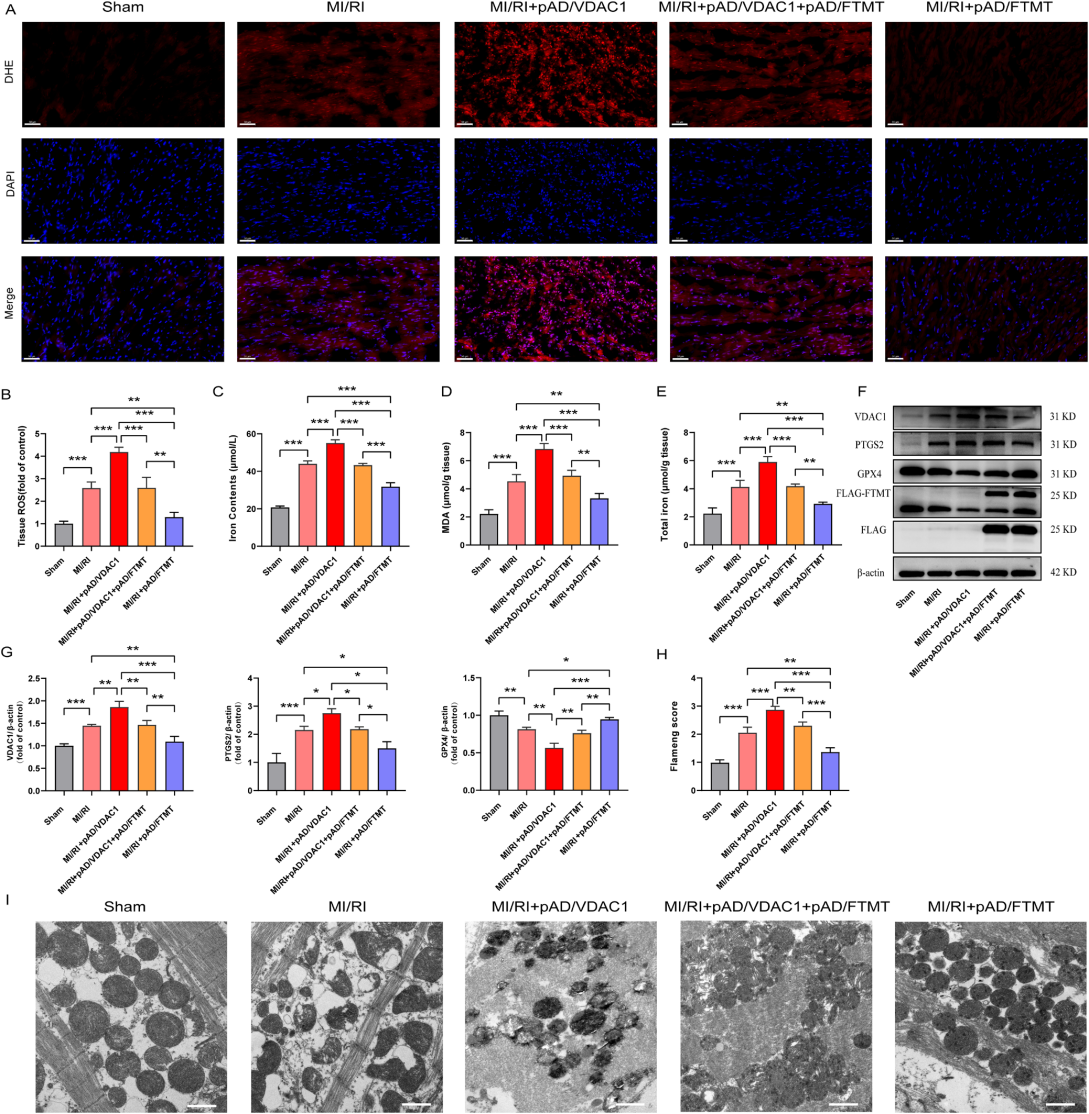
Overexpression of FTMT inhibits MI/RI‐induced ferroptosis and mitochondrial injury by mediating VDAC1. (A, B) Representative images and quantitative data of rat myocardial tissue damage by DHE staining (enlargement, 200×, scaled bar: 50 μm). (C) Serum iron contents. (D, E) Rats heart tissues of MDA, total iron contents. (F, G) Western blot analysis. (H, I) TEM for assessing mitochondrial damage (enlargement, 6500×; scaled bar: 1 μm). Data are presented as the mean ± SD (*n* = 3), **p < 0.01, ***p < 0.001.

To further confirm the role of FTMT and VDAC1 with ferroptosis in MI/RI, we evaluated the effects of pAD/VDAC1 and pAD/FTMT on ferroptosis‐related indices and mitochondrial damage in MI/RI‐induced myocardium. As shown in Figure 9A–E, ROS levels (DHE), iron and MDA content were significantly increased in MI/RI‐treated myocardial tissues or serum and were further elevated after pAD/VDAC1 intervention. In contrast, overexpression of FTMT effectively ameliorated the appealing changes in MI/RI, and further damage to MI/RI by pAD/VDAC1 was significantly blocked by pAD/FTMT treatment. In addition, we also detected the protein expression levels of VDAC1, PTGS2 and GPX4 by Western blot and observed mitochondrial damage in rat myocardial tissue by TEM, and the observations were similar to those of the in vitro experiments (Figure 9F–I). These results suggest that overexpression of FTMT inhibits MI/RI‐induced ferroptosis and mitochondrial damage by mediating VDAC1.

## 4 Discussion

**FIGURE 10:**
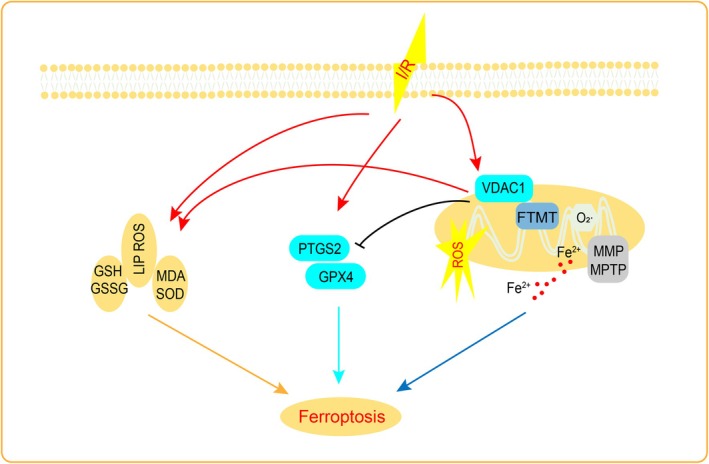
Potential mechanism of VDAC1‐FTMT in MI/RI. Mitochondrial ferritin overexpression attenuates ferroptosis and mitochondrial dysfunction by regulating VDAC1 to relieve myocardium fromMI/RI‐induced damage.

In this work, we investigated the mechanism of action of VDAC1 and FTMT in MI/RI. (1) MI/RI treatment activated VDAC1 and ferroptosis. (2) Overexpression of VDAC1 aggravated ferroptosis, whereas overexpression of FTMT effectively alleviated MI/RI injury by hindering VDAC1 activation and ferroptosis. (3) FTMT overexpression can improve mitochondrial dysfunction by regulating VDAC1. In brief, our study is the first to demonstrate that FTMT overexpression alleviates ferroptosis and mitochondrial dysfunction by regulating VDAC1, thereby reducing MI/RI injury (Figure 10).

Reperfusion injury frequently represents a significant obstacle to the prognostic benefit of ICM patients, particularly in the context of AMI [38]. There are complex programmed regulatory mechanisms behind MI/RI, including apoptosis, autophagy and necrosis [39, 40]. It is therefore essential to conduct a comprehensive examination of the pathophysiological mechanisms underlying MI/RI. Ferroptosis represents a novel form of PCD that occurs in a lipid‐peroxidative, iron‐dependent manner, and mitochondria alterations [41]. The ferroptosis pathway has been demonstrated to be involved in MI/RI. Cyclosporine A ameliorates MI/RI by inhibiting ferroptosis [42]. Naringenin has been observed to mediate the NRF2/System Xc‐/GPX4 signalling pathway, thereby inhibiting ferroptosis and alleviating MI/RI [43]. It has been reported that the glutathione (GSH) system in ferroptosis represents a significant antioxidant defence against ROS. Furthermore, the GSH system is largely dependent on the catalysing effect of glutathione peroxidase (GPX) for the conversion of GSH to GSSG in redox reactions [44]. Consistent with this, our study demonstrated that the GSH system was disrupted and ferroptosis was activated following A/R treatment. Additionally, the ferroptosis‐associated positive/negative (PTGS2/GPX4) marker exhibited a significant elevation/decrease, and VDAC1 expression was significantly activated.

Reperfusion has been demonstrated to result in additional myocardial injury, which may involve the opening of the MPTP. The opening of the MPTP and the ensuing oxidative stress caused by ROS are thought to be a significant mechanism underlying myocardial dysfunction and mitochondrial injury [45]. VDAC1 is a key component of MPTP [13]. In addition to serving as the gatekeeper of MPTP, VDAC1 interacts with TSPO to regulate mitochondrial mass [46]. Excessive opening of MPTP may result in an imbalance in the exchange of substances between mitochondria and the cytoplasm, potentially leading to MMP depolarisation, mitochondrial swelling and fragmentation [47, 48, 49]. Prior research has demonstrated that VDAC1 overexpression impedes the protective effects of resveratrol on MI/RI. Conversely, the downregulation of VDAC1 was observed to effectively attenuate iron overload, thereby alleviating MI/RI‐associated cell death [18]. In our study, VDAC1 overexpression was observed to exacerbate MPTP opening and MMP depolarisation, ROS burst and ferrous iron deposition, GPX4 and FTMT inactivation, resulting in irreversible mitochondrial damage and the promotion of ferroptosis. Conversely, the silencing of VDAC1 was observed to effectively inhibit ferroptosis, thereby ameliorating myocardial injury. Therefore, the development of targeted agents against inhibition of VDAC1 could be a potential strategy for the treatment of MI/RI.

Mitochondria play a pivotal role in the pathogenesis of MI/RI with ferroptosis [32]. Mitochondria can regulate ferroptosis by modulating ROS, and they can also restrict ferroptosis by modulating iron ion metabolism. Despite containing only approximately one‐fifth of the total cellular iron, mitochondria are integral to iron metabolism [50]. Furthermore, FTMT is a mitochondrial protein that stores excess iron ions within the mitochondria, a process that is unique to this organelle [51]. Previous studies have indicated that FTMT may exert a protective effect against cerebral ischaemia–reperfusion/injury by inhibiting apoptosis and ferroptosis [26, 27]. Furthermore, FTMT upregulation has been demonstrated to confer protection against acute force injury in mice [52]. The present study demonstrated that FTMT overexpression effectively reduced MI/RI‐induced iron deposition, ROS and lipid metabolite bursts; this is consistent with previously reported studies that targeted the inhibition of mitochondrial iron overload or ROS accumulation by the overexpression of FTMT, which significantly inhibited oxidative stress‐induced ferroptosis [53]. Although ferritin heavy chain (FTH) upregulation also does not alter the viability of cardiac spheres, the overexpression of FTH significantly inhibits myocardial infarct size [54]. Other studies have shown that myocardial ferritin heavy chain deletion does not alter mitochondrial regulatory and surveillance pathways (fission and fusion) or mitochondrial bioenergetics but protects the heart from MI/RI by activating the expression of anti‐ferroptosis‐related genes [55]. The present study indicates that FTMT not only fulfils the function of storing excess mitochondrial iron but also exhibits antioxidant enzyme activity. Additionally, the overexpression of FTMT has been demonstrated to increase GPX4 levels by downregulating VDAC1 expression. Furthermore, the overexpression of FTMT was observed to improve mitochondrial dysfunction by inhibiting the over‐opening of the MPTP, preventing MMP depolarisation, and maintaining mitochondrial integrity through the down‐regulation of VDAC1 activity. In conclusion, the results of this study indicate that FTMT overexpression can mitigate myocardial injury from MI/RI injury by regulating VDAC1, thereby improving ferroptosis and mitochondrial dysfunction.

### 4.1 Limitations

In the present study, first, we used H9c2 cells that have myogenic properties, can differentiate into a cardiomyocyte‐like phenotype and express cardiac‐specific markers such as troponin and myosin to mimic MI/RI in vitro. This makes H9c2 cells an important tool for studying cardiac physiology and pathology, including ischaemia–reperfusion injury, drug screening and the molecular mechanisms of cardiac disease. However, there are some limitations to the use of H9c2 cells, including the fact that H9c2 cells retain some features of skeletal muscle cells, which may limit their ability to replicate the adult cardiomyocyte phenotype fully, and that H9c2 cells may exhibit differences in contractility, mitochondrial function and fatty acid oxidation compared with primary cardiomyocytes. Second, only male rats were used in our study as in vivo MI/RI. Whether potential differences in body weight and physiological responses in female rats would affect the results is unknown. Second, using only male animals may limit the generalisability of our findings to the female population. In addition, our study did not spatially resolve ferroptosis within border and remote regions. To enhance translational relevance, subsequent research should integrate complementary experimental approaches: (1) Validation of critical findings using primary cardiomyocyte cultures or advanced human‐induced pluripotent stem cell‐derived cardiomyocytes (hiPSC‐CMs) with enhanced electrophysiological maturity; (2) implementation of three‐dimensional engineered cardiac microtissues to better recapitulate myocardial tissue architecture and cell–matrix interactions; (3) systematic evaluation of sex‐dependent molecular mechanisms through comparative studies in age‐matched male and female animal models. Such multidimensional approaches would strengthen the mechanistic understanding of MI/RI while improving the clinical translatability of therapeutic strategies; (4) future studies using spatial transcriptomics or multiplex immunofluorescence could address this gap.

## Author Contributions

Y.Y. performed cell experiments, data analysis, visualisation and original draft. X.W. conducted the cell experiments and data processing. H.X. and L.L. contributed to the methodology. All authors have read and approved the final manuscript. J.L. and S.L. designed the study and revised the manuscript. H.H. supervised the study and confirmed the authenticity of all the raw data.

## Conflicts of Interest

The authors declare no conflicts of interest.

## Supporting information


**FIG. S1** Western blotting of the target protein VDAC1 or FTMT after in vitro experimental adenoviral transduction. (A) VDAC1, (B) FTMT‐FLAG.

**FIG. S2** Silencing VDAC1 alleviated A/R‐induced ferroptosis. (A, B). Western blotting of the target protein VDAC1 after si‐VDAC1 transduction. (C) Assessment of MDA in A/R‐induced after si‐VDAC1 pre‐treatment, (D) SOD; (E)GSH/GSSG ratio; (F–G) Expression of VDAC1, FTMT, PTGS2, and GPX4 proteins; (H and I) ROS levels (enlargement, ×200; scaled bar: 100 μm); (J, K) FerroOrange probe for detecting ferrous ions (enlargement, 200×; scaled bar: 100 μm). Data are presented as the mean ± SD (*n* = 3), **p < 0.01, ***p < 0.001.

**FIG. S3** Successful adenovirus transduction was verified in vivo by western blotting. (A) pAD/VDAC1, (B) pAD/FTMT.

**Table S1.** The sequence.

## Data Availability

Data will be made available on request.

## References

[1] I. Cabac‐Pogorevici , B. Muk , Y. Rustamova , A. Kalogeropoulos , S. Tzeis , and P. Vardas , “Ischaemic Cardiomyopathy. Pathophysiological Insights, Diagnostic Management and the Roles of Revascularisation and Device Treatment. Gaps and Dilemmas in the Era of Advanced Technology,” European Journal of Heart Failure 22, no. 5 (2020): 789–799. 32020756 10.1002/ejhf.1747

[2] M. G. Del Buono , F. Moroni , R. A. Montone , L. Azzalini , T. Sanna , and A. Abbate , “Ischemic Cardiomyopathy and Heart Failure After Acute Myocardial Infarction,” Current Cardiology Reports 24, no. 10 (2022): 1505–1515. 35972638 10.1007/s11886-022-01766-6 PMC9556362

[3] A. Schäfer , T. König , J. Bauersachs , and M. Akin , “Novel Therapeutic Strategies to Reduce Reperfusion Injury After Acute Myocardial Infarction,” Current Problems in Cardiology 47, no. 12 (2022): 101398. 36108813 10.1016/j.cpcardiol.2022.101398

[4] X. J. Zhang , X. Liu , M. Hu , et al., “Pharmacological Inhibition of Arachidonate 12‐Lipoxygenase Ameliorates Myocardial Ischemia‐Reperfusion Injury in Multiple Species,” Cell Metabolism 33, no. 10 (2021): 2059–2075.e10. 34536344 10.1016/j.cmet.2021.08.014

[5] X. Jia , W. Shao , and S. Tian , “Berberine Alleviates Myocardial Ischemia‐Reperfusion Injury by Inhibiting Inflammatory Response and Oxidative Stress: The Key Function of miR‐26b‐5p‐Mediated PTGS2/MAPK Signal Transduction,” Pharmaceutical Biology 60, no. 1 (2022): 652–663. 35311466 10.1080/13880209.2022.2048029 PMC8967400

[6] L. Li , L. Lin , S. Lei , S. Shi , C. Chen , and Z. Xia , “Maslinic Acid Inhibits Myocardial Ischemia‐Reperfusion Injury‐Induced Apoptosis and Necroptosis via Promoting Autophagic Flux,” DNA and Cell Biology 41, no. 5 (2022): 487–497. 35475713 10.1089/dna.2021.0918

[7] S. J. Dixon , K. M. Lemberg , M. R. Lamprecht , et al., “Ferroptosis: An Iron‐Dependent Form of Nonapoptotic Cell Death,” Cell 149, no. 5 (2012): 1060–1072. 22632970 10.1016/j.cell.2012.03.042 PMC3367386

[8] X. Jiang , B. R. Stockwell , and M. Conrad , “Ferroptosis: Mechanisms, Biology and Role in Disease,” Nature Reviews. Molecular Cell Biology 22, no. 4 (2021): 266–282. 33495651 10.1038/s41580-020-00324-8 PMC8142022

[9] X. Wu , Y. Li , S. Zhang , and X. Zhou , “Ferroptosis as a Novel Therapeutic Target for Cardiovascular Disease,” Theranostics 11, no. 7 (2021): 3052–3059. 33537073 10.7150/thno.54113 PMC7847684

[10] J. Yan , Z. Li , Y. Liang , et al., “Fucoxanthin Alleviated Myocardial Ischemia and Reperfusion Injury Through Inhibition of Ferroptosis via the NRF2 Signaling Pathway,” Food & Function 14, no. 22 (2023): 10052–10068. 37861458 10.1039/d3fo02633g

[11] J. P. Friedmann Angeli , M. Schneider , B. Proneth , et al., “Inactivation of the Ferroptosis Regulator Gpx4 Triggers Acute Renal Failure in Mice,” Nature Cell Biology 16, no. 12 (2014): 1180–1191. 25402683 10.1038/ncb3064 PMC4894846

[12] C. Ge , Y. Peng , J. Li , et al., “Hydroxysafflor Yellow A Alleviates Acute Myocardial Ischemia/Reperfusion Injury in Mice by Inhibiting Ferroptosis via the Activation of the HIF‐1α/SLC7A11/GPX4 Signaling Pathway,” Nutrients 15, no. 15 (2023): 3411. 37571350 10.3390/nu15153411 PMC10420812

[13] A. Camara , Y. Zhou , P. C. Wen , E. Tajkhorshid , and W. M. Kwok , “Mitochondrial VDAC1: A Key Gatekeeper as Potential Therapeutic Target,” Frontiers in Physiology 8 (2017): 460. 28713289 10.3389/fphys.2017.00460 PMC5491678

[14] V. Shoshan‐Barmatz , V. De Pinto , M. Zweckstetter , Z. Raviv , N. Keinan , and N. Arbel , “VDAC, a Multi‐Functional Mitochondrial Protein Regulating Cell Life and Death,” Molecular Aspects of Medicine 31, no. 3 (2010): 227–285. 20346371 10.1016/j.mam.2010.03.002

[15] G. Tian , J. Zhou , Y. Quan , et al., “Voltage‐Dependent Anion Channel 1 (VDAC1) Overexpression Alleviates Cardiac Fibroblast Activation in Cardiac Fibrosis via Regulating Fatty Acid Metabolism,” Redox Biology 67 (2023): 102907. 37797372 10.1016/j.redox.2023.102907 PMC10622884

[16] Z. Liao , D. Liu , L. Tang , et al., “Long‐Term Oral Resveratrol Intake Provides Nutritional Preconditioning Against Myocardial Ischemia/Reperfusion Injury: Involvement of VDAC1 Downregulation,” Molecular Nutrition & Food Research 59, no. 3 (2015): 454–464. 25488258 10.1002/mnfr.201400730

[17] Y. Feng , N. B. Madungwe , A. D. Imam Aliagan , N. Tombo , and J. C. Bopassa , “Liproxstatin‐1 Protects the Mouse Myocardium Against Ischemia/Reperfusion Injury by Decreasing VDAC1 Levels and Restoring GPX4 Levels,” Biochemical and Biophysical Research Communications 520, no. 3 (2019): 606–611. 31623831 10.1016/j.bbrc.2019.10.006 PMC7457545

[18] T. Hu , H. X. Zou , Z. Y. Zhang , et al., “Resveratrol Protects Cardiomyocytes Against Ischemia/Reperfusion‐Induced Ferroptosis via Inhibition of the VDAC1/GPX4 Pathway,” European Journal of Pharmacology 971 (2024): 176524. 38561102 10.1016/j.ejphar.2024.176524

[19] T. Hu , H. X. Zou , S. Y. Le , et al., “Tanshinone IIA Confers Protection Against Myocardial Ischemia/Reperfusion Injury by Inhibiting Ferroptosis and Apoptosis via VDAC1,” International Journal of Molecular Medicine 52, no. 5 (2023): 109. 37800609 10.3892/ijmm.2023.5312 PMC10558218

[20] S. Levi and P. Arosio , “Mitochondrial Ferritin,” International Journal of Biochemistry & Cell Biology 36, no. 10 (2004): 1887–1889. 15203103 10.1016/j.biocel.2003.10.020

[21] B. Corsi , A. Cozzi , P. Arosio , et al., “Human Mitochondrial Ferritin Expressed in HeLa Cells Incorporates Iron and Affects Cellular Iron Metabolism,” Journal of Biological Chemistry 277, no. 25 (2002): 22430–22437. 11953424 10.1074/jbc.M105372200

[22] S. Levi , B. Corsi , M. Bosisio , et al., “A Human Mitochondrial Ferritin Encoded by an Intronless Gene,” Journal of Biological Chemistry 276, no. 27 (2001): 24437–24440. 11323407 10.1074/jbc.C100141200

[23] P. Santambrogio , G. Biasiotto , F. Sanvito , S. Olivieri , P. Arosio , and S. Levi , “Mitochondrial Ferritin Expression in Adult Mouse Tissues,” Journal of Histochemistry and Cytochemistry 55, no. 11 (2007): 1129–1137. 17625226 10.1369/jhc.7A7273.2007 PMC3957534

[24] Z. H. Shi , F. F. Shi , Y. Q. Wang , et al., “Mitochondrial Ferritin, a New Target for Inhibiting Neuronal Tumor Cell Proliferation,” Cellular and Molecular Life Sciences 72, no. 5 (2015): 983–997. 25213357 10.1007/s00018-014-1730-0 PMC4323545

[25] P. Wang , Q. Ren , M. Shi , Y. Liu , H. Bai , and Y. Z. Chang , “Overexpression of Mitochondrial Ferritin Enhances Blood‐Brain Barrier Integrity Following Ischemic Stroke in Mice by Maintaining Iron Homeostasis in Endothelial Cells,” Antioxidants (Basel) 11, no. 7 (2022): 1257. 35883748 10.3390/antiox11071257 PMC9312053

[26] P. Wang , Y. Cui , Q. Ren , et al., “Mitochondrial Ferritin Attenuates Cerebral Ischaemia/Reperfusion Injury by Inhibiting Ferroptosis,” Cell Death & Disease 12, no. 5 (2021): 447. 33953171 10.1038/s41419-021-03725-5 PMC8099895

[27] P. Wang , Y. Cui , Y. Liu , et al., “Mitochondrial Ferritin Alleviates Apoptosis by Enhancing Mitochondrial Bioenergetics and Stimulating Glucose Metabolism in Cerebral Ischemia Reperfusion,” Redox Biology 57 (2022): 102475. 36179435 10.1016/j.redox.2022.102475 PMC9526171

[28] F. Sun , C. An , C. Liu , et al., “FTO Represses NLRP3‐Mediated Pyroptosis and Alleviates Myocardial Ischemia‐Reperfusion Injury via Inhibiting CBL‐Mediated Ubiquitination and Degradation of β‐Catenin,” FASEB Journal 37, no. 6 (2023): e22964. 37199660 10.1096/fj.202201793RR

[29] H. He , Y. Luo , Y. Qiao , et al., “Curcumin Attenuates Doxorubicin‐Induced Cardiotoxicity via Suppressing Oxidative Stress and Preventing Mitochondrial Dysfunction Mediated by 14‐3‐3γ,” Food & Function 9, no. 8 (2018): 4404–4418. 30063064 10.1039/c8fo00466h

[30] F. Hu , T. Hu , A. He , et al., “Puerarin Protects Myocardium From Ischaemia/Reperfusion Injury by Inhibiting Ferroptosis Through Downregulation of VDAC1,” Journal of Cellular and Molecular Medicine 28, no. 24 (2024): e70313. 39730981 10.1111/jcmm.70313 PMC11680193

[31] Y. Yuan , S. Lai , T. Hu , et al., “Puerarin Pretreatment Provides Protection Against Myocardial Ischemia/Reperfusion Injury via Inhibiting Excessive Autophagy and Apoptosis by Modulation of HES1,” Scientific Reports 15, no. 1 (2025): 794. 39755744 10.1038/s41598-024-84808-z PMC11700218

[32] T. Hu , W. P. Yu , X. Q. Wang , et al., “Activation of PPAR‐α Attenuates Myocardial Ischemia/Reperfusion Injury by Inhibiting Ferroptosis and Mitochondrial Injury via Upregulating 14‐3‐3η,” Scientific Reports 14, no. 1 (2024): 15246. 38956068 10.1038/s41598-024-64638-9 PMC11219969

[33] T. Hu , F. J. Hu , H. Huang , et al., “Epigallocatechin‐3‐Gallate Confers Protection Against Myocardial Ischemia/Reperfusion Injury by Inhibiting Ferroptosis, Apoptosis, and Autophagy via Modulation of 14‐3‐3η,” Biomedicine & Pharmacotherapy 174 (2024): 116542. 38574620 10.1016/j.biopha.2024.116542

[34] J. Guo , S. B. Wang , T. Y. Yuan , et al., “Coptisine Protects Rat Heart Against Myocardial Ischemia/Reperfusion Injury by Suppressing Myocardial Apoptosis and Inflammation,” Atherosclerosis 231, no. 2 (2013): 384–391. 24267256 10.1016/j.atherosclerosis.2013.10.003

[35] X. T. Li , X. Y. Li , T. Tian , et al., “The UCP2/PINK1/LC3b‐Mediated Mitophagy Is Involved in the Protection of NRG1 Against Myocardial Ischemia/Reperfusion Injury,” Redox Biology 80 (2025): 103511. 39874927 10.1016/j.redox.2025.103511 PMC11808529

[36] S. Zhang , Y. Ye , Q. Li , et al., “Andrographolide Attenuates Myocardial Ischemia‐Reperfusion Injury in Mice by up‐Regulating PPAR‐α,” Inflammation (2024). 10.1007/s10753-024-02193-1 39585583

[37] W. Flameng , M. Borgers , W. Daenen , and G. Stalpaert , “Ultrastructural and Cytochemical Correlates of Myocardial Protection by Cardiac Hypothermia in Man,” Journal of Thoracic and Cardiovascular Surgery 79, no. 3 (1980): 413–424. 6243726

[38] S. M. Davidson , P. Ferdinandy , I. Andreadou , et al., “Multitarget Strategies to Reduce Myocardial Ischemia/Reperfusion Injury: JACC Review Topic of the Week,” Journal of the American College of Cardiology 73, no. 1 (2019): 89–99. 30621955 10.1016/j.jacc.2018.09.086

[39] D. Liu , H. Wu , Y. Z. Li , et al., “Cellular FADD‐Like IL‐1β‐Converting Enzyme‐Inhibitory Protein Attenuates Myocardial Ischemia/Reperfusion Injury via Suppressing Apoptosis and Autophagy Simultaneously,” Nutrition, Metabolism, and Cardiovascular Diseases 31, no. 6 (2021): 1916–1928. 10.1016/j.numecd.2021.02.026 33895078

[40] G. Zhou , H. Wu , J. Yang , et al., “Liraglutide Attenuates Myocardial Ischemia/Reperfusion Injury Through the Inhibition of Necroptosis by Activating GLP‐1R/PI3K/Akt Pathway,” Cardiovascular Toxicology 23, no. 3–4 (2023): 161–175. 36934206 10.1007/s12012-023-09789-3

[41] X. Luan , P. Chen , L. Miao , X. Yuan , C. Yu , and G. Di , “Ferroptosis in Organ Ischemia‐Reperfusion Injuries: Recent Advancements and Strategies,” Molecular and Cellular Biochemistry 480 (2024): 19–41. 38556592 10.1007/s11010-024-04978-2

[42] W. Qian , D. Liu , Y. Han , et al., “Cyclosporine A‐Loaded Apoferritin Alleviates Myocardial Ischemia‐Reperfusion Injury by Simultaneously Blocking Ferroptosis and Apoptosis of Cardiomyocytes,” Acta Biomaterialia 160 (2023): 265–280. 36822483 10.1016/j.actbio.2023.02.025

[43] S. Xu , B. Wu , B. Zhong , et al., “Naringenin Alleviates Myocardial Ischemia/Reperfusion Injury by Regulating the Nuclear Factor‐Erythroid Factor 2‐Related Factor 2 (Nrf2) /System xc−/ Glutathione Peroxidase 4 (GPX4) Axis to Inhibit Ferroptosis,” Bioengineered 12, no. 2 (2021): 10924–10934. 34699317 10.1080/21655979.2021.1995994 PMC8809912

[44] F. Ursini and M. Maiorino , “Lipid Peroxidation and Ferroptosis: The Role of GSH and GPx4,” Free Radical Biology & Medicine 152 (2020): 175–185. 32165281 10.1016/j.freeradbiomed.2020.02.027

[45] C. Penna , M. G. Perrelli , and P. Pagliaro , “Mitochondrial Pathways, Permeability Transition Pore, and Redox Signaling in Cardioprotection: Therapeutic Implications,” Antioxidants & Redox Signaling 18, no. 5 (2013): 556–599. 22668069 10.1089/ars.2011.4459

[46] J. Gatliff , D. East , J. Crosby , et al., “TSPO Interacts With VDAC1 and Triggers a ROS‐Mediated Inhibition of Mitochondrial Quality Control,” Autophagy 10, no. 12 (2014): 2279–2296. 25470454 10.4161/15548627.2014.991665 PMC4502750

[47] D. Lin , B. Cui , J. Ren , and J. Ma , “Regulation of VDAC1 Contributes to the Cardioprotective Effects of Penehyclidine Hydrochloride During Myocardial Ischemia/Reperfusion,” Experimental Cell Research 367, no. 2 (2018): 257–263. 29630893 10.1016/j.yexcr.2018.04.004

[48] G. Chanoit , J. Zhou , S. Lee , et al., “Inhibition of Phosphodiesterases Leads to Prevention of the Mitochondrial Permeability Transition Pore Opening and Reperfusion Injury in Cardiac H9c2 Cells,” Cardiovascular Drugs and Therapy 25, no. 4 (2011): 299–306. 21643720 10.1007/s10557-011-6310-z

[49] L. Gomez , B. Li , N. Mewton , et al., “Inhibition of Mitochondrial Permeability Transition Pore Opening: Translation to Patients,” Cardiovascular Research 83, no. 2 (2009): 226–233. 19221132 10.1093/cvr/cvp063

[50] A. M. Battaglia , R. Chirillo , I. Aversa , A. Sacco , F. Costanzo , and F. Biamonte , “Ferroptosis and Cancer: Mitochondria Meet the “Iron Maiden” Cell Death,” Cells 9, no. 6 (2020): 1505. 32575749 10.3390/cells9061505 PMC7349567

[51] Y. Song , M. Gao , B. Wei , et al., “Mitochondrial Ferritin Alleviates Ferroptosis in a Kainic Acid‐Induced Mouse Epilepsy Model by Regulating Iron Homeostasis: Involvement of Nuclear Factor Erythroid 2‐Related Factor 2,” CNS Neuroscience & Therapeutics 30, no. 3 (2024): e14663. 38439636 10.1111/cns.14663 PMC10912846

[52] W. Wu , S. Chang , Q. Wu , et al., “Mitochondrial Ferritin Protects the Murine Myocardium From Acute Exhaustive Exercise Injury,” Cell Death & Disease 7, no. 11 (2016): e2475. 27853170 10.1038/cddis.2016.372 PMC5260894

[53] Y. Chen , X. Guo , Y. Zeng , et al., “Oxidative Stress Induces Mitochondrial Iron Overload and Ferroptotic Cell Death,” Scientific Reports 13, no. 1 (2023): 15515. 37726294 10.1038/s41598-023-42760-4 PMC10509277

[54] M. Campan , V. Lionetti , G. D. Aquaro , et al., “Ferritin as a Reporter Gene for In Vivo Tracking of Stem Cells by 1.5‐T Cardiac MRI in a Rat Model of Myocardial Infarction,” American Journal of Physiology. Heart and Circulatory Physiology 300, no. 6 (2011): H2238–H2250. 21335465 10.1152/ajpheart.00935.2010

[55] S. E. Machado , D. Spangler , D. A. Stacks , et al., “Counteraction of Myocardial Ferritin Heavy Chain Deficiency by Heme Oxygenase‐1,” International Journal of Molecular Sciences 23, no. 15 (2022): 8300. 35955444 10.3390/ijms23158300 PMC9368247

