# Supplementary figures and images for: Mitochondrial Ferritin Overexpression Attenuates Ferroptosis and Mitochondrial Dysfunction by Reducing VDAC1 to Relieve MI/RI‐Induced Damage
Source: J Cell Mol Med. 2025 Jun 23;29(12):e70650. doi: 10.1111/jcmm.70650 (PMC12185369; doi:10.1111/jcmm.70650)

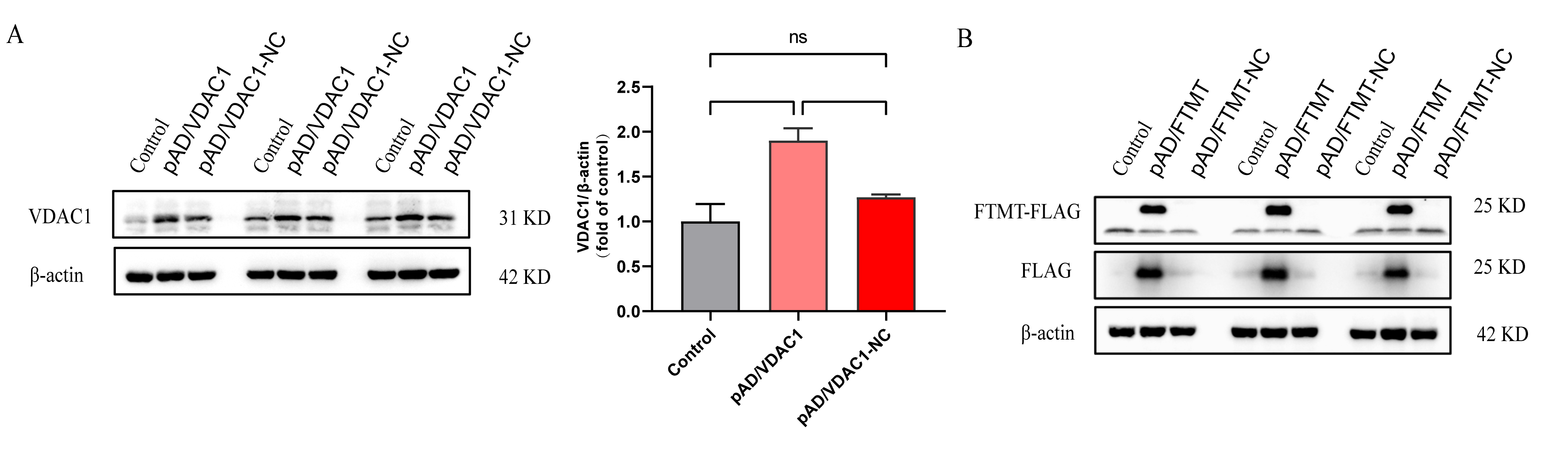

Supplement: Supplementary file 1 — FIG. S1 Western blotting of the target protein VDAC1 or FTMT after in vitro experimental adenoviral transduction. (A) VDAC1, (B) FTMT‐FLAG. [file JCMM-29-e70650-s004.png]

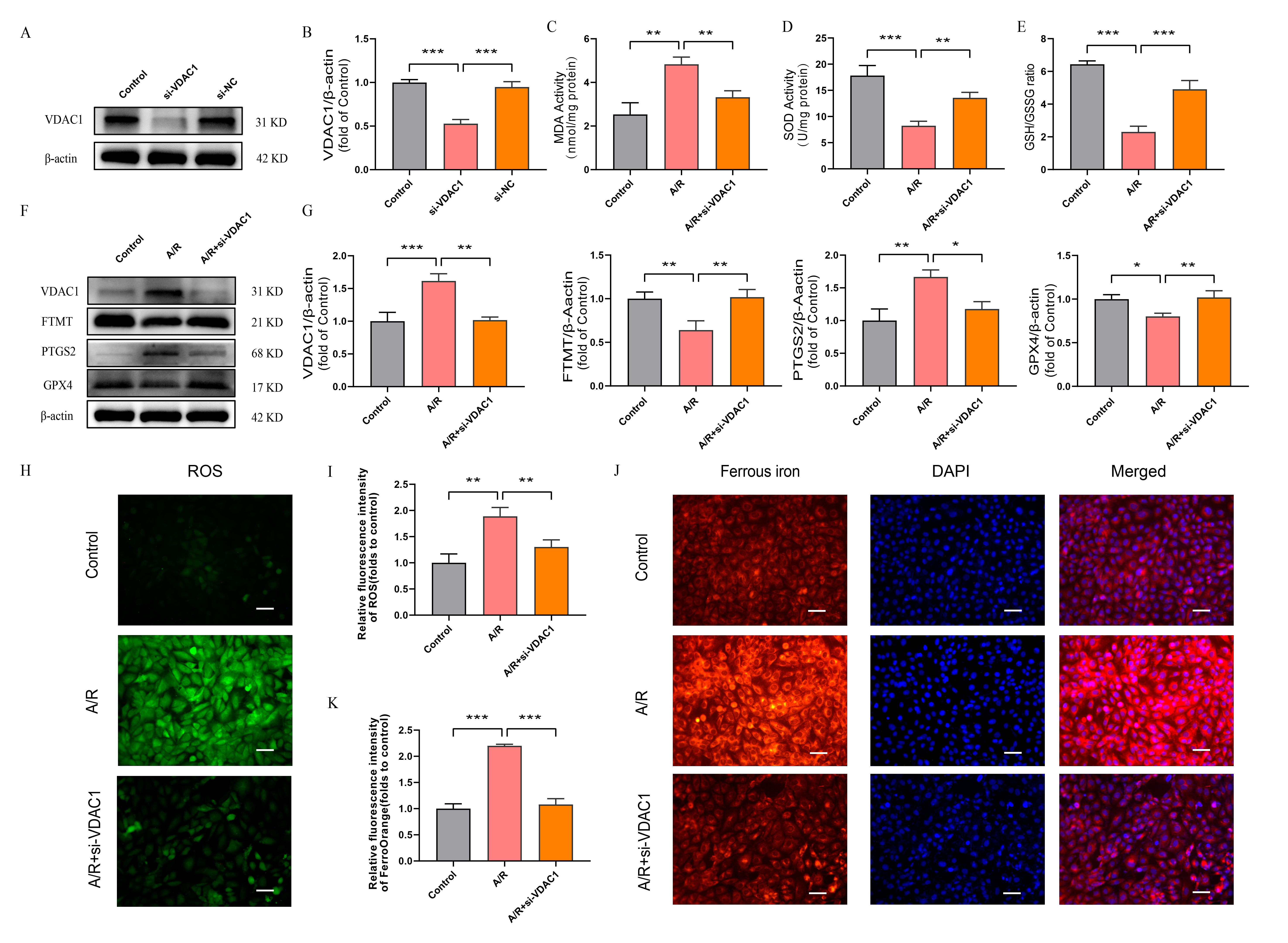

Supplement: Supplementary file 2 — FIG. S2 Silencing VDAC1 alleviated A/R‐induced ferroptosis. (A, B). Western blotting of the target protein VDAC1 after si‐VDAC1 transduction. (C) Assessment of MDA in A/R‐induced after si‐VDAC1 pre‐treatment, (D) SOD; (E)GSH/GSSG ratio; (F–G) Expression of VDAC1, FTMT, PTGS2, and GPX4 proteins; (H and I) ROS levels (enlargement, ×200; scaled bar: 100 μm); (J, K) FerroOrange probe for detecting ferrous ions (enlargement, 200×; scaled bar: 100 μm). Data are presented as the mean ± SD (n = 3), **p < 0.01, ***p < 0.001. [file JCMM-29-e70650-s001.png]

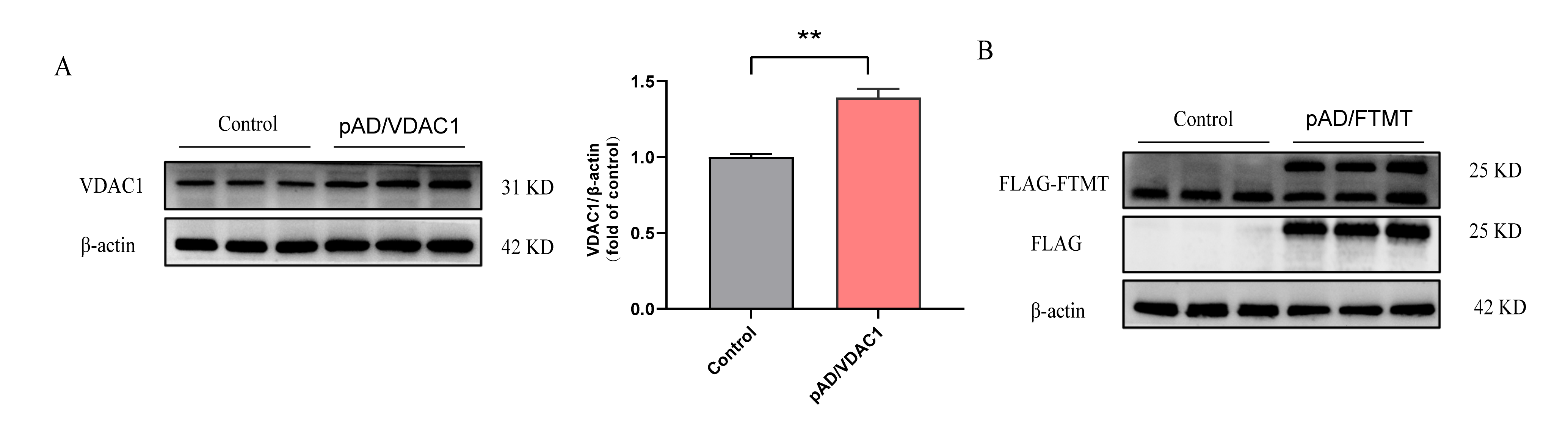

Supplement: Supplementary file 3 — FIG. S3 Successful adenovirus transduction was verified in vivo by western blotting. (A) pAD/VDAC1, (B) pAD/FTMT. [file JCMM-29-e70650-s003.png]
